# Hydrogen Sulfide Inhibits *H. pylori*-Induced Gastric Fibroblast Activation: Implications for Cancer Prevention

**DOI:** 10.3390/cells15020167

**Published:** 2026-01-16

**Authors:** Gracjana Krzysiek-Maczka, Aneta Targosz, Patrycja Bronowicka-Adamska, Urszula Szczyrk, Malgorzata Strzalka, Hubert Mączka, Mateusz Wierdak, Izabela Rodzon, Jaroslaw Czyz, Tomasz Brzozowski, Agata Ptak-Belowska

**Affiliations:** 1 Department of Physiology, Faculty of Medicine, Jagiellonian University Medical College, Grzegorzecka 16, 31-531 Cracow, Poland; aneta.targosz@uj.edu.pl (A.T.); urszula.szczyrk@uj.edu.pl (U.S.); malgorzata.strzalka@uj.edu.pl (M.S.); izabela.ksiazek@uj.edu.pl (I.R.); agata.ptak-belowska@uj.edu.pl (A.P.-B.); 2 Department of Medical Biochemistry, Faculty of Medicine, Jagiellonian University Medical College, Mikolaja Kopernika 7c, 31-034 Cracow, Poland; patrycja.bronowicka-adamska@uj.edu.pl; 3 Student Research Group, Department of Physiology, Faculty of Medicine, Jagiellonian University Medical College, Grzegorzecka 16, 31-531 Cracow, Poland; hubert.maczka@student.uj.edu.pl; 4 Department of Endoscopic, Metabolic and Soft Tissue Malignancies Surgery of University Hospital, Jagiellonian University, Jakubowskiego 2, 30-688 Cracow, Poland; mateusz.wierdak@uj.edu.pl; 5 Department of Cell Biology, Faculty of Biochemistry, Biophysics and Biotechnology, Jagiellonian University, Gronostajowa 7, 30-387 Cracow, Poland; jarek.czyz@uj.edu.pl

**Keywords:** Helicobacter pylori-activated fibroblasts, gastric cancer associated fibroblasts, NF-κB, STAT3, hydrogen sulfide (H_2_S)

## Abstract

**Highlights:**

**What are the main findings?**

- *Hp* (*cagA^+^vacA^+^*)-induced inflammation reduces gastric fibroblast proliferation/viability and reprograms these cells toward a CAF-like phenotype (Twist upregulation/nuclear localization, increased *FAP/FSP*, enhanced *IL-6/IL-8/HGF* expression), upregulation of NF-κB/STAT3 activation and induction of the sulfur-metabolizing enzymes CBS, MPST and TST.
- The fast-releasing H_2_S donor NaHS (50–400 µM) attenuates *Hp*-induced fibroblast activation markers/secretome, reduces Twist and NF-κB/STAT3 activation, and modulates the sulfur/H_2_S-metabolizing enzyme network without disrupting basal enzyme homeostasis.

**What is the implication of the main finding?**

- Based on our data, modulation of H_2_S signaling may be considered as a possible approach to attenuate *Hp*-dependent fibroblast activation and to help maintain fibroblast homeostasis and, at higher NaHS doses, can additionally reduce *Hp* adhesion and increase metronidazole sensitivity.
- Preliminary experiments with the slow-releasing donor GYY4137 (50–100 µM) support follow-up studies using slow-release H_2_S donors to better approximate physiological H_2_S signaling.

**Abstract:**

Early prevention of pathological changes underlying gastric cancer (GC) development is a critical strategy, offering the most effective opportunity to limit malignant progression and improve patient outcomes. We have previously demonstrated that *Helicobacter pylori* (*Hp*) (*cagA^+^vacA*^+^) contributes to GC development by activating gastric fibroblasts toward CAF-like phenotype, eliciting aggressive, cancer stem cells (CSCs)-related malignant transformation of LGR5^+^ normal epithelial cells. A key mediator of these processes appears to be the NF-κB/STAT3 axis. Therefore, our aim was to investigate the protective role of hydrogen sulfide (H_2_S) as a potential novel strategy for counteracting *Hp*-induced fibroblast reprogramming. Human fibroblasts were infected with *Hp* (*cagA+vacA+*) for 120 h. The fast-releasing H_2_S donor NaHS (50, 100, 200 and 400 µM) was added every 24 h. Activation markers, corresponding signaling pathways, H_2_S release and activities of H_2_S-metabolizing enzymes were determined. NaHS reduced *Hp*-induced fibroblast activation and their pro-inflammatory, pro-tumorigenic markers, which was associated with the inhibition of NF-κB/STAT3 axis and Twist expression. Additionally, it modulated sulfur metabolism while preserving sulfur-enzyme homeostasis. NaHS limited *Hp* adhesion (high doses), reduced reinfection-induced activation and increased sensitivity of *Hp* to metronidazole. These findings suggest that H_2_S signaling may represent a modulatory factor of NF-κB/STAT3-driven inflammatory responses during *Hp* infection and warrant further investigation.

## 1. Introduction

Over the past few decades, the incidence and mortality rates of gastric cancer (GC) have declined [1] due to an improved understanding of epidemiology, molecular mechanisms, clinical diagnosis, and treatment [2]. Nevertheless, GC remains the fifth-most common cancer and the third leading cause of cancer-related death worldwide, with 90% of all stomach tumors being malignant [1,2,3]. Despite advances in early detection and treatment strategies including surgery, chemotherapy, radiotherapy, and targeted therapy, patients still experience GC recurrence and metastasis [1]. This challenge is partly attributed to the involvement of multiple stem/progenitor cells that can serve as a source of cancer stem cells driving tumor initiation, progression, therapy resistance, and recurrence after treatment and remission [1,2]. Although treatment of disseminated GC may alleviate symptoms and modestly prolong survival, long-term remissions remain rare, emphasizing the need for further analysis of GC pathogenesis, particularly regarding major pathogens [1,2]. GC development is a complex process involving genetic and epigenetic alterations of oncogenes, cell cycle regulators, and signaling molecules [4,5]. These alterations can be promoted by gastric infection with the Gram-negative bacterium *Helicobacter pylori* (*Hp*), which has been classified by the WHO as a Class I carcinogen [2,3]. *Hp*-induced GC development is primarily characterized by the interplay among *Hp* virulence factors, host factors, and environmental influences that culminate in chronic inflammation. This inflammatory process is accompanied by the release of various signaling molecules including cytokines, chemokines, and growth factors (GFs) that stimulate the homing and activation of cellular effectors, such as fibroblasts, thereby promoting the formation of cancer stem cells, the establishment of their niches, and ultimately GC development [3,4,6,7,8]. Central to this process are *Hp* virulence factors such as cytotoxin-associated gene A (CagA), delivered into host cells via the type IV secretion system, and pathogen-associated molecular pattern recognition by pattern-recognition receptors (PRRs), including Toll-like receptors (TLRs). Through these inputs, *Hp* persistently activates NF-κB and STAT3 signaling in gastric epithelial and stromal compartments, thereby integrating inflammatory and stress cues into transcriptional programs that support malignant transformation and tumor progression [3,4,6,7,8,9,10,11,12]. We have previously demonstrated that *Hp* (*cagA^+^vacA^+^*) activates normal rat and human gastric fibroblasts, toward cells possessing characteristics of cancer-associated fibroblasts (CAFs) able to elicit reprogramming of normal rat gastric epithelial LGR5-positive cells toward epithelial–mesenchymal transition type 3 (EMT-3)-related pro-pluripotent, carcinogenic and invasive phenotype reflecting the flexibility of cancer stem cells (CSCs) [4,5,6,7,8]. Furthermore, *Hp*-activated fibroblasts accelerated the invasiveness of AGS and HT29 cancer cells in vitro [8]. These findings underscore the role of fibroblast activation towards a CAF phenotype in the neoplastic reorganization of the gastric niche, which, in simplified terms, may guide the promotion, progression and invasiveness of GC, particularly of the lethal diffuse type. Consistent with this concept, CAFs are now recognized as major organizers of the tumor microenvironment (TME), where they modulate ECM composition and stiffness, sustain chronic inflammation via secretion of IL-6, IL-8, HGF and other mediators, and promote angiogenesis, immune evasion and metastatic dissemination [4,7,8,9,10,11,12,13,14,15]. Given that CAFs play a pivotal role in the modulation of the TME, targeting these cells may reduce tumor-promoting events by blocking tumor development, proliferation, and metastasis. Moreover, considering the often-late detection of *Hp* infection and the increasing antibiotic resistance of *Hp*, it is imperative to investigate strategies to halt and possibly reverse *Hp*-induced fibroblast activation. In recent years, the role of hydrogen sulfide (H_2_S) in regulating the physiology and pathophysiology of the digestive system, including cancer, has attracted significant attention [16,17,18,19,20,21]. H_2_S is a ubiquitous gaseous signaling molecule produced endogenously via precisely regulated enzymatic and non-enzymatic desulfhydration. It is primarily generated by cystathionine β-synthase (CBS), cystathionine γ-lyase (CSE), and 3-mercaptopyruvate sulfurtransferase (3-MST), with CBS and CSE considered the main sources [8,10,11]. Non-enzymatic H_2_S production can also occur through the reduction in sulfane sulfur species (SSS) [16]. In humans, H_2_S is either metabolized directly or stored in the form of bound sulfane sulfur (SS) and acid-labile sulfur [16,17]. SSS are suggested to mediate some of the biological functions of H_2_S, representing an intracellular storage pool that can release H_2_S upon reaction with reducing agents. Currently, it is believed that SSS are the main products of H_2_S oxidation and that their abundance correlates with H_2_S-synthesizing activity. Additionally, oxidative degradation of H_2_S by the sulfur oxidation pathway (SOP) enzymes in the mitochondria further contributes to maintaining H_2_S homeostasis [17,19,20,21,22]. TST, a key enzyme in the oxidation and metabolism of H_2_S, reflects the cell’s ability to convert exogenous H_2_S into bound sulfur compounds, which is crucial for maintaining redox homeostasis and stress resistance. The regulation of TST activity occurs at multiple levels, including dose-dependent H_2_S-induced persulfidation [16,17,21,22]. Thus, the balance between H_2_S administration/synthesis and its degradation is essential to prevent cytotoxicity, and disturbances in these enzymatic activities have been implicated in various pathological disorders [16,17,18,19,21]. Both exogenous H_2_S and the activation of endogenous H_2_S biosynthesis have been shown to prevent altered differentiation, cellular senescence and increased inflammation [23,24,25,26,27]. H_2_S and its derived sulfur species act as signaling molecules via two major mechanisms: binding to protein metal centers, particularly heme iron, and modifying oxidized cysteine residues in proteins, leading to either activation or inhibition of protein function [16,19,22]. Consequently, H_2_S influences gene expression through various mechanisms, including epigenetic modifications and transcriptional regulation. Exogenous H_2_S is among others postulated as an effective inhibitor of the NFκB pathway, blocking its activation and nuclear translocation, and consequently reducing the transcription of pro-inflammatory genes and the production of cytokines such as IL-8 and IL-6, which are the common link between the NF-κB and STAT3 pathways [16,17,19,22,25,28,29,30]. In addition, H_2_S has been reported to activate the Nrf2 pathway via Keap1 S-sulfhydration, thereby enhancing antioxidant and cytoprotective responses (e.g., HO-1, Trx, GST, GPx, TrxR) and reducing ROS that would otherwise sustain NF-κB/STAT3 signaling [30,31,32,33,34,35]. Together with accumulating evidence that CBS, MPST and TST can support redox buffering, metabolic reprogramming and stress resistance in tumors [16,17,18,19], these findings position H_2_S and sulfur metabolism at the intersection of inflammatory, redox and stromal-remodeling pathways that are all engaged during *Hp*-driven gastric carcinogenesis. Dysregulation of NF-κB activation and the JAK/STAT3 signaling pathway is observed in many cancers, including GC, and correlates with disease progression and poor prognosis. These pathways regulate numerous genes involved in cell differentiation, proliferation, angiogenesis, apoptosis, and cancer-related inflammation, thereby promoting a malignant state [28,36,37]. The interplay between STAT3 and NF-κB is particularly critical in mediating crosstalk between malignant cells and the TME. For instance, cytokines and growth factors induced in response to NF-κB in the TME can activate STAT3 in both malignant and stromal cells, establishing a positive feedback loop that reinforces their oncogenic activities [13,15,31,38]. The downstream targets of NF-κB/STAT3 include genes involved in reprogramming (e.g., *Snail1*, *Twist*, *Zeb*), immunosuppression, proliferation, survival, angiogenesis and metastasis [4,5,6,7]. Accordingly, STAT3/NF-κB signaling has been implicated not only in tumor stemness, chemoresistance and metastasis in GC, but also in persistent activation of stromal fibroblasts [6,14,15,17,31,38]. In this study, we sought to elucidate the modulatory effect of exogenous H_2_S on *Hp*-induced activation of normal human gastric fibroblasts (NHGF). Our goal was to explore the protective mechanism of H_2_S as a potential novel strategy for treating *Hp*-related gastric disease by modulating fibroblast activation and the pro-inflammatory microenvironment, either as a complement or, in selected contexts, an alternative to conventional anti-*Hp* therapy, particularly considering the challenges posed by late detection, increased antibiotic resistance and side effects of current medications.

## 2. Materials and Methods

### 2.1. Bacterial Strain and Culture

Hp strain 43504 (*cagA+vacA+* (*s1/m1*); ATCC, Manassas, VA, USA) was cultured on Columbia Agar with 5% fresh horse blood (BioMérieux, Marcy-l’Étoile, France) under microaerophilic conditions at 37 °C for 3–5 days. Stock cultures were maintained at −70 °C in Brucella Broth (Becton Dickinson, Franklin Lakes, NT, USA) supplemented with 10% FBS and 10% glycerol. Prior to co-incubation, bacteria were resuspended in DMEM supplemented with 10% FBS [6,7,8]. *Hp* strain ATCC 43504 (*cagA+vacA+* (*s1/m1*)) was selected as the reference high-virulence strain based on genotyping and functional screening of the *H. pylori* isolates in our collection. Using PCR amplification of *cagA* and *vacA* alleles, we identified two strains carrying the high-virulence genotype [39] *cagA+vacA (s1/m1)* (Supplementary Figure S2). In contrast, strains with the *cagA−vacA−* (*s2/m2*) genotype did not activate gastric fibroblasts in our earlier work [40]. Among the two *cagA+vacA+* (*s1/m1*) strains (ATCC 43504 and 700824), ATCC 43504 reproducibly elicited the strongest fibroblast-activating response in our assays, including the highest induction of Twist and pro-inflammatory cytokines. For this reason, ATCC 43504 was used in all experiments in the present study (Supplementary Figure S2).

### 2.2. Fibroblast Culture

Primary gastric fibroblasts were obtained from gastric mucosa biopsies (Hp-negative patients without systemic inflammatory and autoimmunologic diseases undergoing laparoscopic sleeve gastrectomy) by collagenase P digestion (Sigma-Aldrich, Saint Louis, MO, USA). Secondary cultures were established by trypsinization. Cells were cultured in DMEM + 10% FBS and antibiotics at 37 °C, 5% CO_2_. Primary gastric fibroblasts used in this study were obtained from two to three independent human donors. For selected experiments, non-gastric BJ fibroblasts (CRL-2522, ATCC, Manassas, VA, USA) were included to assess whether Hp-induced activation and NaHS responsiveness were consistent across fibroblast backgrounds. All cultures were screened for mycoplasma using MycoCheck™ PCR kit (MoBiTech GmbH, Göttingen, Germany).

### 2.3. In Vitro Hp Infection Model

Subconfluently, fibroblasts were infected twice with 1 × 10^9^ live *Hp* per dish and incubated for 48–120 h. Media volume and handling procedures were standardized across experiments [6,7,8].

### 2.4. H_2_S Donor Treatment

Non-infected and *Hp*-infected fibroblasts were treated daily with the H_2_S fast-releasing donor NaHS (50–400 µM) (Sigma-Aldrich, Saint Louis, MO, USA) for 96 and 120 h in DMEM + 10%FBS with no addition of antibiotics. To assess whether the effects observed with NaHS reflected H_2_S-dependent activity rather than donor-specific properties, *Hp*-infected fibroblasts were additionally treated with the slow-releasing H_2_S donor GYY4137 (50 and 100 µM; Cayman Chemical, Ann Arbor, MI, USA) for 96 h. GYY4137 (Sigma-Aldrich, Saint Louis, MO, USA) was added daily under the same culture conditions as above.

Control NaCl Treatment: To exclude nonspecific effects related to Na^+^ or osmolarity, fibroblasts were treated daily with NaCl (Sigma-Aldrich, Saint Louis, MO, USA) (50 and 400 µM) for 96 h under identical culture conditions.

### 2.5. Immunofluorescence Imaging

Cells were fixed with acetone: methanol, permeabilized with 0.1% Triton X-100, and incubated with anti-Twist antibody (ab50581, Abcam, Cambridge, UK). Secondary antibody: mouse anti-rabbit IgG (F4890, Sigma-Aldrich, Saint Louis, MO, USA). Nuclei were counterstained with Hoechst 33,258 (B2883, Sigma-Aldrich, Saint Louis, MO, USA). Image acquisition and processing were performed using Leica DMI6000B microscope and LAS X 3.4 software (Leica, Wetzlar, Germany). 

### 2.6. RT-qPCR

Fibroblasts (8 × 10^6^/well) were cultured for 96 h. Total RNA was isolated with TRIzol (Invitrogen, Thermo Fisher Scientific, Carlsbad, CA, USA) [6], quantified with Nanodrop ND-1000, and reverse-transcribed using Promega A3500 kit. Expression for Twist, IL-6, STAT3, IL-8, FAP, HGF, CBS, MPST, TST, Ki67 was determined using specific primers (Sigma Aldrich, St. Louis, MO, USA). Primer sequences are listed in Table 1. The expression of reference *18S* RNA was used for the RNA integrity verification. PCR was conducted on Rotor Gene RG-3000 using GoTaq^®^ Master Mix (Promega Corporation, Madison, WI, USA). Melt curves were analyzed to ensure specificity. Relative expression was calculated using ΔΔCt method [41]. Primer sequences are listed in Table S1.

### 2.7. Western Blot (WB)

Proteins were extracted using Subcellular Protein Fractionation Kit (Thermo Fisher Scientific, Waltham, MA, USA), quantified by Nanodrop. Proteins were separated by SDS-PAGE (NuPAGE, Invitrogen, Thermo Fisher Scientific, Carlsbad, CA, USA), transferred to nitrocellulose membranes, and probed with the following antibodies: anti-GAPDH, p-IKKα, p-IKKβ, p-p65, p-STAT3, CBS, MPST. The primary antibodies used: anti-GAPDH (D16H11 Cell Signaling Technology, Inc., Danvers, MA, USA), anti-p-IKKα (PA5121282, Invitrogen, Thermo Fisher Scientific, Carlsbad, CA, USA), anti-p-IKKβ (PA5-36653 Invitrogen, Thermo Fisher Scientific, Carlsbad, CA, USA), anti-p-p65 and anti-p-STAT3 (GTX118000 GeneTex, Inc., Irvine, CA, USA), anti-CBS (NBP315413, Novus Biologicals, LLC, Centennial, CO, USA) and anti-MPST (GTX108274, GeneTex, Inc., Irvine, CA, USA). The HRP-conjugated secondary antibody used was goat antirabbit IgG (ab97051 Abcam, Cambridge, UK). Bands were visualized by chemiluminescence and quantified using Image Studio Lite software ver. 5.5 (LI-COR Biosciences, Lincoln, NE, USA).

### 2.8. H_2_S Production and Enzyme Activity

H_2_S production in bacterial strain pellets was examined essentially as described in previous studies [42]. The results were expressed as µmoles/mg of protein. To precisely determine the difference in H_2_S release between *Hp*-infected and non-infected fibroblasts, 7-Azido-4-Methylcoumarin (AzMC) probe (Sigma-Aldrich, St. Louis, MO, USA) was used. Fluorescence was measured at 355/460 nm on a Synergy HTX plate reader (BioTek Instruments, Inc., Winooski, VT, USA) [43].

### 2.9. SS Content

SS content was determined by the Wood method according to the detailed procedure and the protein content was determined by Lowry method [44]. Values for infected fibroblasts were normalized to the cell count (adjusted comparing to the control).

### 2.10. H_2_S-Metabolizing Enzymes Activity in Fibroblasts

CSE activity was determined using Matsuo and Greenberg’s method with modifications [45]. MPST activity was evaluated by Valentine and Frankelfeld method [46]. TST activity was assayed by Sorbo’s method, with modification [47]. Values of enzymes activities for infected fibroblasts were normalized to the cell number (adjusted comparing to the control). The concentration of CBS was measured by CBS ELISE (Kit Cat. No. E3445Hu, Bioassay Technology Laboratory, Shanghai, China). The measurement was performed using a microplate reader Epoch (Instruments, Inc., Winooski, VT, USA). Values for infected fibroblasts were normalized to the cell number (adjusted comparing to the control).

### 2.11. Cell Viability

Fibroblasts (4 × 10^4^/well) were exposed to *Hp* ± NaHS. Viability was assessed by ReadyCount™ Green/Red stain (Invitrogen, Thermo Fisher Scientific, Carlsbad, CA, USA) using Countess3FL (Invitrogen, Thermo Fisher Scientific, Carlsbad, CA, USA) and validated with Trypan Blue staining (Sigma-Aldrich, St. Louis, MO, USA) in Burker chamber (BRAND GmbH + Co. KG, Wertheim, Germany).

### 2.12. The Sensitivity of Hp to Metronidazole

MBC (Minimum Bactericidal Concentration) of metronidazole for *Hp* (43504 Hp *cagA+vacA+* (*s1/m1*)) strain have been determined by liquid broth micro-dilution as previously described [48]. MBC was defined as the lowest concentration of the drug that killed 99.9% of the starting CFU (0.1% CFU survival). The metronidazole was administered at doses of 8, 50, 60, 80, 85, 90, 95, 100, 105, 110, 115 and 120 mg/L for 48 h. The same procedure was applied to assess the influence of 50 μM, 100 μM, 200 μM and 400 μM NaHS (Merck KGaA, Darmstadt, Germany) on the growth and viability of *Hp* as well as for the assessment of the influence of combined doses of metronidazole and NaHS on the MBC values.

### 2.13. Statistical Analysis

Data is presented as mean ± SEM. Statistical significance was evaluated using one-way ANOVA followed by Newman–Keuls or Tukey post hoc tests. *p* < 0.05 was considered significant.

## 3. Results

### 3.1. Effect of NaHS on Hp-Induced Human Gastric Fibroblast Activation—The Involvement of Proinflammatory Pathways in the Fibroblast Reprogramming

**Figure 1:**
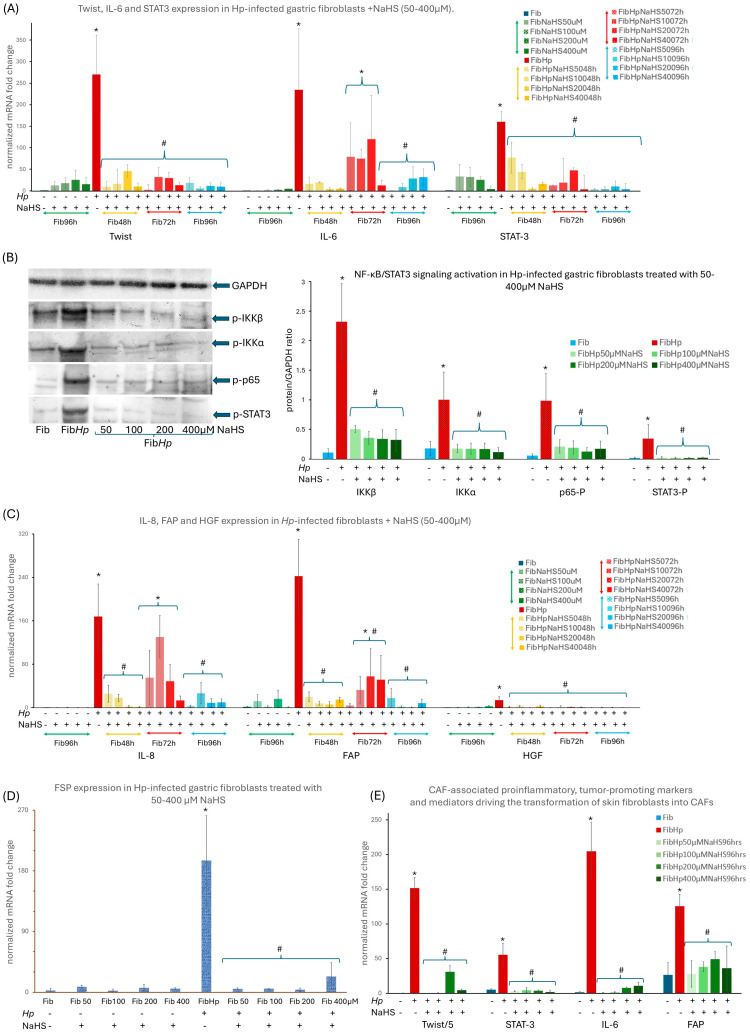
NaHS attenuates Hp-induced activation of gastric fibroblasts and CAF-associated inflammatory signaling. All analyses were performed in non-infected and *Hp*-infected normal human gastric fibroblasts (NHGFs) treated with NaHS (50–400 µM); no antibiotics were included. qPCR analysis of selected transcriptional and signaling mediators involved in *Hp*-induced fibroblast activation toward a CAF-like phenotype (*Twist*, *IL-6*, *STAT3*) measured over 96 h. The panel illustrates the time- and dose-dependent modulation of *Hp*-induced gene expression by NaHS (**A**). Western blot analysis of NF-κB/STAT3 pathway activation after 120 h of culture. Phosphorylated IKKα, IKKβ, p65, and STAT3 were analyzed in total cellular protein extracts. Right panel shows semi-quantitative densitometric analysis normalized to GAPDH (10 µg protein loaded per lane) (**B**). qPCR analysis of CAF-associated pro-inflammatory and tumor-promoting markers (*IL-8*, *FAP*, *HGF*) in *Hp*-infected NHGFs treated with NaHS for 96 h, illustrating attenuation of the *Hp*-induced CAF-like secretory profile (**C**). qPCR analysis of *FSP* mRNA expression in *Hp*-infected NHGFs following 96 h NaHS treatment (**D**). qPCR analysis of CAF-associated markers and activating mediators in BJ normal human skin fibroblasts after 96 h of *Hp* infection and NaHS treatment, demonstrating that the observed modulatory effects are not restricted to gastric fibroblasts. For visualization purposes, *Twist* and *IL-6* values were divided by 5 to allow comparison with lower-abundance transcripts (**E**). All qPCR data were normalized to *18S* rRNA and are presented as fold change relative to non-infected fibroblasts. Results are mean ± SEM of two to six independent experimental repeats. Asterisk (*) indicates a significant change (*p* < 0.05) as compared to the control value (non-infected fibroblasts). Hash (#) indicates a significant change (*p* < 0.05) as compared to *Hp*-infected fibroblasts.

**Figure 2:**
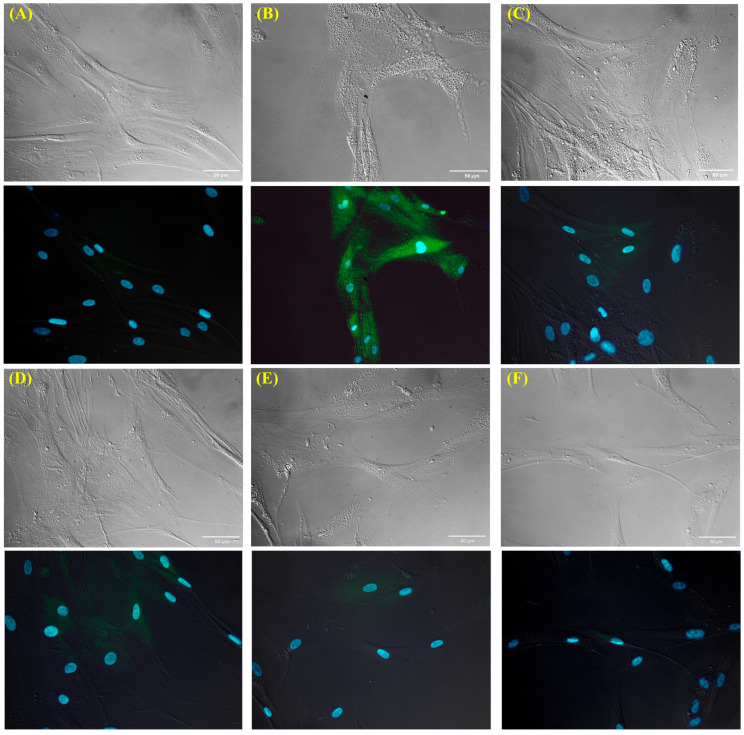
NaHS abolishes Hp-induced Twist protein expression and nuclear localization in gastric fibroblasts. Nomarski contrast and composite images of immunofluorescence staining for chromatin and Twist protein in NHGFs: non-infected (**A**) *Hp*-infected (**B**) and of *Hp*-infected NHGFs treated with NaHS for 96 h at increasing concentrations: 50 µM (**C**), 100 µM (**D**), 200 µM (**E**), and 400 µM (**F**). No antibiotics were added. The images demonstrate the progressive disappearance of Twist protein and its nuclear localization in *Hp*-infected NHGFs upon NaHS administration (sequentially: Nomarski contrast and composite images). Green: Twist protein; Blue: chromatin (Hoechst 33258).

**Figure 3:**
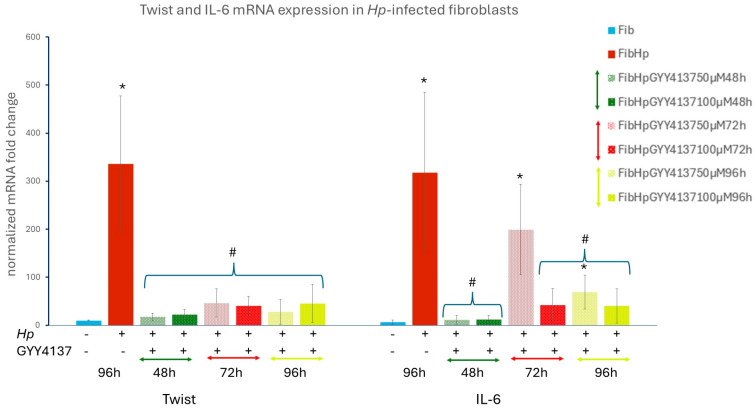
Effect of the slow-releasing H_2_S donor GYY4137 on *Twist* and *IL-6* mRNA expression in Hp-infected gastric fibroblasts resulting in a reduction in *Hp*-induced *Twist* and *IL-6* expression. qPCR analysis of *Twist* and *IL-6* mRNA expression in non-infected fibroblasts (Fib), *Hp*-infected fibroblasts (Fib*Hp*), and *Hp*-infected fibroblasts treated with GYY4137 (50 or 100 µM) for 48, 72 and 96 h. Gene expression was normalized to *18S* rRNA and is presented as fold change relative to non-infected fibroblasts. All qPCR data were normalized to *18S* rRNA and are presented as fold change relative to non-infected fibroblasts. Results are mean ± SEM of four independent experimental repeats. Asterisk (*) indicates a significant change (*p* < 0.05) as compared to the control value (non-infected fibroblasts). Hash (#) indicates a significant change (*p* < 0.05) as compared to *Hp*-infected fibroblasts.

In accordance with our previous findings [6,7,8], *Hp* infection strongly reprogrammed gastric fibroblasts toward a CAF-like phenotype. *Hp*-infected NHGFs showed a robust induction of the CAF marker transcription factor *Twist*, *IL-6* and *STAT3* mRNA (Figure 1A), accompanied by elevated activation markers *FAP* (Figure 1C) and *FSP* (Figure 1D) mRNAs together with a proinflammatory, tumor-promoting secretome [9,10,11,12,13] enriched in *IL-8* and *HGF* (Figure 1C). The appearance of Twist protein in *Hp*-infected gastric fibroblasts and its nuclear localization was confirmed by fluorescent staining (Figure 2B). These changes coincided with strong activation of NF-κB and STAT3 signaling, as reflected by enhanced phosphorylation of IKKα, IKKβ, p65 and STAT3 (Figure 1B). Given the central role of NF-κB and STAT3 in *Hp*-associated gastric carcinogenesis and TME, remodeling and our observation that these pathways are robustly activated in *Hp*-infected gastric fibroblasts, we next evaluated whether NaHS, a donor of H_2_S, could attenuate fibroblast activation and modulate the accompanying NF-κB/STAT3 signaling cascade. We have used NaHS at 50–400 µM, a concentration range widely applied to model acute, transient H_2_S exposure in mammalian cells [49], and NaHS treatment at these levels markedly suppressed *Hp*-induced fibroblast activation. To verify that NaHS did not exert nonspecific physicochemical effects under our experimental conditions, we estimated its influence on medium composition. The highest concentration used (400 µM) can increase medium osmolarity by <1 mOsm and Na^+^ levels by ~0.4 mM relative to DMEM (≈290–330 mOsm/L; 130–155 mM Na^+^), i.e., <0.3% of baseline values, so it is far below the range typically required to affect mammalian cells [50,51,52]. Nevertheless, to ensure that no such effect occurred, we treated fibroblasts with 50 and 400 µM NaCl for 96 h. The fibroblasts showed no significant changes in *Twist* or *IL-6* mRNA expression (Supplementary Figure S1) which provides strong evidence that NaHS-associated effects observed in *Hp*-infected fibroblasts are not attributable to Na^+^ or osmolarity variations. Moreover, NaHS did not induce statistically significant changes in the mRNA expression of CAF proinflammatory and tumor-promoting markers (Figure 1A,C,D). Next, we have shown that administration of NaHS at concentrations of 50, 100, 200 and 400 μM significantly reduced the level of *Twist*, *IL-6*, *STAT3*, *IL-8*, *FAP* and *HGF* mRNA expression already 48 h after the *Hp* infection (Figure 1A,C). Additionally, NaHS showed protective effect against *Hp* infection as reinfection with *Hp* in the 48th hour of the experimental procedure resulted after next 24 h (72nd h of the experiment) in lower expression of mRNA for *STAT3* (all tested doses), *IL-6* (particularly for 400 μM NaHS) (Figure 1A), *IL-8* (particularly for 200 and 400 µM NaHS) accompanied with significantly decreased fibroblast activation marker *FAP* in all tested doses (Figure 1C). Then the expression of the above factors continued to decrease (96th hour of the experiment) back to the control level (Figure 1A,C,D). Importantly, the reinfection of fibroblasts did not induce any increase either in *Twist* (Figure 1A) or in HGF (all tested doses) (Figure 1C). NaHS in all tested doses also abolished *Hp*-induced Twist protein expression and its nuclear localization, with complete fluorescence loss at 400 μM (Figure 2C–F). After 96 h, all NaHS concentrations reduced *Twist*, *IL-6*, *STAT3*, *IL-8*, *FAP*, *HGF* and *FSP* mRNA expression back to control (non-infected) levels (Figure 1A,C,D), with a corresponding normalization of NF-κB/STAT3 phosphorylation (Figure 1B). Consistently, NaHS in all tested doses attenuated phosphorylation of IKKα, p65 and STAT3 after 120 h of treatment, reducing their levels to values comparable to those observed in non-infected fibroblasts, while p-IKKβ, though strongly suppressed, stayed slightly elevated versus non-infected fibroblasts (Figure 1B). Notably, NaHS fully abolished Hp-induced Twist protein accumulation and nuclear localization, with complete loss of fluorescence at 400 μM (Figure 2C–F). To determine whether this response is tissue specific, normal human skin fibroblasts (BJ) were analyzed in parallel. *Hp* infection activated BJ fibroblasts in a manner analogous to gastric fibroblasts, and NaHS consistently reduced *Twist*, *STAT3*, *IL-6* and *FAP* mRNA expression after 96 h of treatment all tested doses (Figure 1E). Collectively, these results indicate that NaHS substantially attenuates NF-κB/STAT3 activation and suppresses the CAF-associated proinflammatory and tumor-promoting transcriptional program induced by *Hp* in both gastric and skin fibroblasts. In addition, exploratory experiments with the slow-releasing donor GYY4137 (50 and 100 µM, added daily) were performed to verify that the observed effects reflect H_2_S-dependent activity rather than donor-specific properties. GYY4137 reduced *Twist* and *IL-6* mRNA expression in *Hp*-infected fibroblasts, indicating that both fast- and slow-releasing H_2_S donors can attenuate *Hp*-induced fibroblast activation under our conditions (Figure 3). After 48 h, administration of GYY4137 at 50 µM and 100 µM markedly suppressed *Twist* and *IL-6* mRNA levels relative to untreated *Hp*-infected fibroblasts. The magnitude of suppression was comparable between the two concentrations. After 72 h, corresponding to the reinfection time point, a secondary increase in *Twist* and *IL-6* expression was observed in *Hp*-infected fibroblasts. Despite this reinfection-associated induction, treatment with GYY4137 at both 50 µM and 100 µM significantly attenuated the expression of both genes compared with untreated *Hp*-infected cells. At this time point, 100 µM GYY4137 showed a tendency toward stronger suppression, particularly for *IL-6*, although both concentrations remained effective. After 96 h, *Twist* and *IL-6* mRNA expression in GYY4137-treated *Hp*-infected fibroblasts remained significantly lower than in untreated Hp-infected cells, indicating sustained suppression over time. For *Twist*, expression levels were reduced close to those observed in non-infected fibroblasts, whereas *IL-6* expression, although strongly decreased, remained modestly elevated relative to baseline. Overall, both 50 µM and 100 µM GYY4137 effectively suppressed *Hp*-induced *Twist* and *IL-6* expression throughout the experimental time course, including at the reinfection-associated 72 h time point, with no pronounced dose-dependent difference except for a trend toward stronger inhibition of *IL-6* at higher concentration (Figure 3).

### 3.2. The H_2_S Metabolism Engagement in the Activation of Human Gastric Fibroblasts

**Figure 4:**
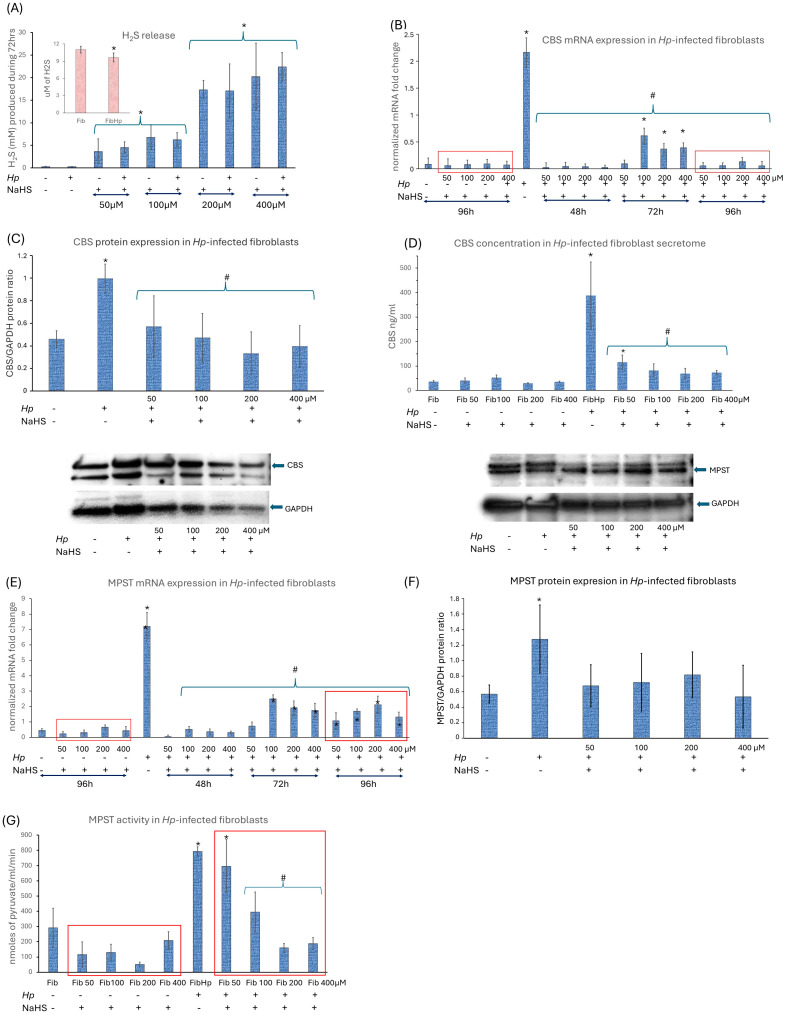
Effects of *Hp* infection and NaHS administration on CBS and MPST expression and activity in gastric fibroblasts. All analysis included were non-infected and *Hp*-infected NHGFs treated with NaHS (50–400 µM). No antibiotics were added. Changes in H_2_S release in non-infected and *Hp*-infected NHGFs following NaHS treatment (50, 100, 200 and 400 µM) measured by trapping H_2_S as zinc sulfide in the zinc agarose layer during 72 h of culture. The upper part of the graph shows more precise differences in H_2_S release between non-infected and *Hp*-infected NHGFs measured with AzMC fluorescent probe after 72 h of culture (**A**). qPCR analysis of the *CBS* mRNA expression (**B**) Elisa analysis of the CBS concentration after 96 h of culture. Values for infected fibroblasts were normalized to cell count (adjusted comparing to the control) (**C**). WB analysis of CBS expression after 120 h of culture. The semi-quantitative densitometry analysis of the ratio of CBS protein over GAPDH. 10 μg of total cellular proteins were loaded per lane (**D**). qPCR analysis of the *MPST* mRNA expression (**E**). WB analysis of MPST expression after 120 h of culture. The semi-quantitative densitometry analysis of the ratio of MPST protein over GAPDH. 10 μg of total cellular proteins were loaded per lane (**F**). MPST activity analysis after 72 h of culture. Values for infected fibroblasts were normalized to cell count (adjusted comparing to the control) and MPST activity is expressed as nmoles of pyruvate produced during 1 min incubation per 1 mL (**G**). qPCR analysis of gene expression was performed after 48, 72, and 96 h of culture. qPCR analyses were normalized to *18S* rRNA and data are presented as fold changes relative to the control group. Results are mean ± SEM of two to six independent experimental repeats. The red boxes indicate the corresponding NaHS-treated groups (50–400 µM) after 96 h of incubation. Asterisk (*) indicates a significant change (*p* < 0.05) as compared to the control value (non-infected fibroblasts). Hash (#) indicates a significant change (*p* < 0.05) as compared to *Hp*-infected fibroblasts.

**Figure 5:**
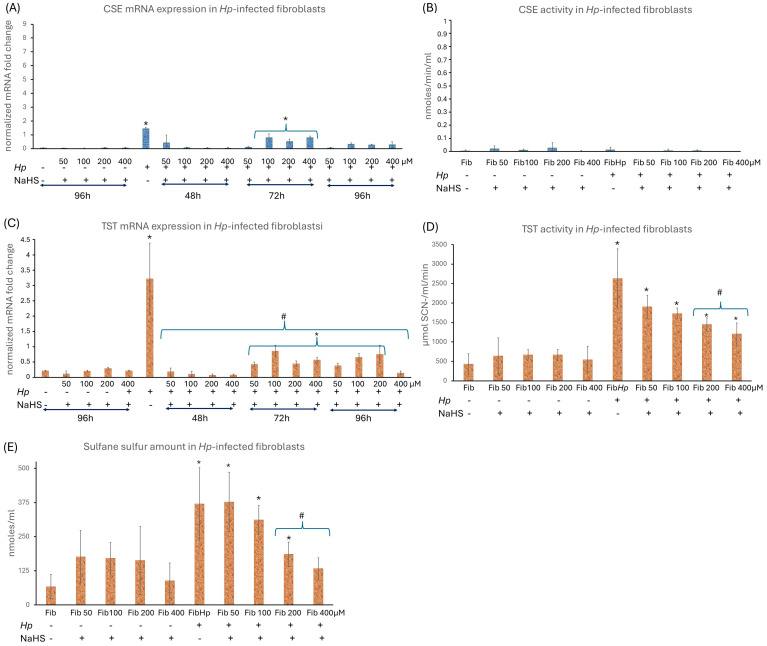
Effects of *Hp* infection and NaHS administration on CSE and TST expression, activity and sulfane sulfur (SS) levels in gastric fibroblasts. All analysis included non-infected and *Hp*-infected NHGFs treated with NaHS (50–400 µM). No antibiotics were added. qPCR analysis of the expression of the *CSE* mRNA during 96 h of culture (**A**). Analysis of CSE activity showing a nearly zero level during *Hp* infection and following NaHS treatment. Values for infected fibroblasts were normalized to the cell count (adjusted comparing to the control). CSE activity is expressed as nmoles of α-ketobutyrate produced during 1 min incubation per 1 mL (**B**). qPCR analysis of the expression of the *TST* mRNA during 96 h of culture (**C**). TST activity was analyzed after 72 h of culture. Values for infected fibroblasts were normalized to cell count (adjusted comparing to the control). TST activity is expressed as nmoles of SCN- formed during 1 min of incubation per 1 mL (**D**). The SS content analysis after 96 h of culture. The SS level was expressed as nmoles of SCN- produced per 1 mL (**E**). qPCR analysis of gene expression was performed after 48, 72, and 96 h of culture. qPCR analyses were normalized to *18S* rRNA and data are presented as fold changes relative to the control group. Results are mean ± SEM of two to six independent experimental repeats. Asterisk (*) indicates a significant change (*p* < 0.05) as compared to the control value (non-infected fibroblasts). Hash (#) indicates a significant change (*p* < 0.05) as compared to *Hp*-infected fibroblasts.

In the first step, we quantified how *Hp* infection and NaHS treatment affect extracellular H_2_S release by NHGFs over 72 h (Figure 4A). Basal H_2_S production measured using zinc-agarose trapping was low and comparable between non-infected and *Hp*-infected fibroblasts (Figure 4A). However, the more sensitive AzMC-based endpoint measurement revealed significantly lower free H_2_S levels in *Hp*-infected versus non-infected cells (Figure 4A, inset), indicating that infection accelerates H_2_S turnover, favoring increased H_2_S enhanced H_2_S turnover and redistribution of H_2_S-derived sulfur into oxidized and protein-bound sulfur species rather than increased net release. In non-infected fibroblasts, NaHS supplementation (50–400 µM) increased extracellular H_2_S compared with untreated controls, with a clear tendency toward higher levels at higher NaHS concentrations (Figure 4A), demonstrating that exogenous NaHS acts as an efficient and titratable H_2_S source in our system. A similar upward trend was observed in *Hp*-infected fibroblasts upon NaHS addition and there was no consistent, statistically significant separation between infected and non-infected fibroblasts at the same NaHS concentrations, indicating comparable capacity to handle exogenous H_2_S (Figure 4A). In our experimental model, H_2_S release in normal, non-infected gastric fibroblasts is primarily driven by CBS and MPST, as confirmed by their mRNA and protein levels and by MPST activity (Figure 4B–G). CSE expression and activity were negligible (Figure 5A,B). *Hp* infection increased CBS mRNA and protein, elevated MPST mRNA and activity, and only slightly induced CSE mRNA without affecting CSE activity (Figure 4B–G and Figure 5A,B). It also upregulated TST mRNA and activity (Figure 5C,D), suggesting that fibroblasts under *Hp* infection parallelly intensify H_2_S production and H_2_S oxidation pathways, particularly TST-mediated sulfur transfer critical for stress tolerance and redox homeostasis [21]. This is consistent with our observation of reduced free H_2_S in *Hp*-infected NHGFs (Figure 4A, inset) and elevated SS content (Figure 5E), supporting the interpretation that *Hp* infection promotes H_2_S production and its metabolism by enhanced H_2_S turnover and redistribution of H_2_S-derived sulfur into oxidized and protein-bound sulfur species. Taken together, these changes suggest an adaptive, potentially protective mechanism that enables fibroblasts to cope with inflammatory and oxidative stress and to reprogram toward a CAF-like phenotype under *Hp*-driven conditions. The addition of NaHS at all tested doses reduced *CBS*, *MPST* and *TST* mRNA expression back to control level of non-infected fibroblasts already after 48 h of administration (Figure 4B,E and Figure 5C). The *Hp* reinfection induced a much lower increase in *CBS* and *MPST* mRNA expression across all NaHS concentrations (72nd hour of experiment) (Figure 4B,E). The reinfection showed also only a small increase in *TST* mRNA (Figure 5C). NaHS administration (50–400 μM) in non-infected fibroblasts did not significantly alter CBS, MPST, CSE, or TST mRNA, protein or activity (Figure 4B,D,E,G and Figure 5A–D). A minor decrease in MPST activity (notably at 200 μM) was observed, without affecting TST activity (Figure 4G and Figure 5D). SS levels showed a slight but statistically insignificant increase at 50–200 μM NaHS and returned to baseline at 400 μM (Figure 5E), suggesting that exogenous H_2_S induces only minor physiological modulation in uninfected cells. The addition of NaHS to infected fibroblasts reduced CBS and MPST mRNA and protein levels (Figure 4B–F), although *MPST* mRNA expression remained elevated after reinfection up to 96 h. MPST activity remained elevated at 50 μM NaHS but decreased to baseline at higher doses (Figure 4G). NaHS also reduced *TST* mRNA expression, which stayed minimally elevated except for 400 μM NaHS (Figure 5C). TST activity was lower in NaHS-treated *Hp*-infected fibroblasts than in infected cells without NaHS, showing a tendency toward greater reduction at higher NaHS doses, with significant effects at 200 and 400 μM (Figure 5D). Notably, despite this decrease, TST activity in *Hp*-infected fibroblasts treated with NaHS remained moderately elevated compared to non-infected fibroblasts, which may reflect an adaptive mechanism whereby fibroblasts maintain TST activity to manage oxidative stress and preserve redox balance under *Hp*-associated stress. Upon NaHS administration in *Hp*-infected cells, SS levels for 50 and 100 μM did not significantly change compared to infected fibroblasts without NaHS administration. Significant reductions appeared at 200 and 400 μM NaHS but there was an overall trend toward declining SS with increasing NaHS concentrations (Figure 5E). These findings suggest that while exogenous H_2_S downregulates endogenous H_2_S-producing enzymes in *Hp*-infected fibroblasts, it does not further increase SS storage, suggesting complex feedback regulation within the H_2_S metabolic network rather than linear additive effects. Together, these data indicate that CBS and MPST are primary drivers of basal H_2_S biosynthesis, whereas *Hp* infection induces coordinated upregulation of both H_2_S-producing and H_2_S-oxidizing pathways, shifting sulfur metabolism toward enhanced turnover. NaHS administration modifies this infection-induced program, predominantly by dampening endogenous H_2_S enzyme induction without amplifying SS accumulation.

### 3.3. The Cytoprotective Role of NaHS in Hp-Induced Fibroblast Infection

**Figure 6:**
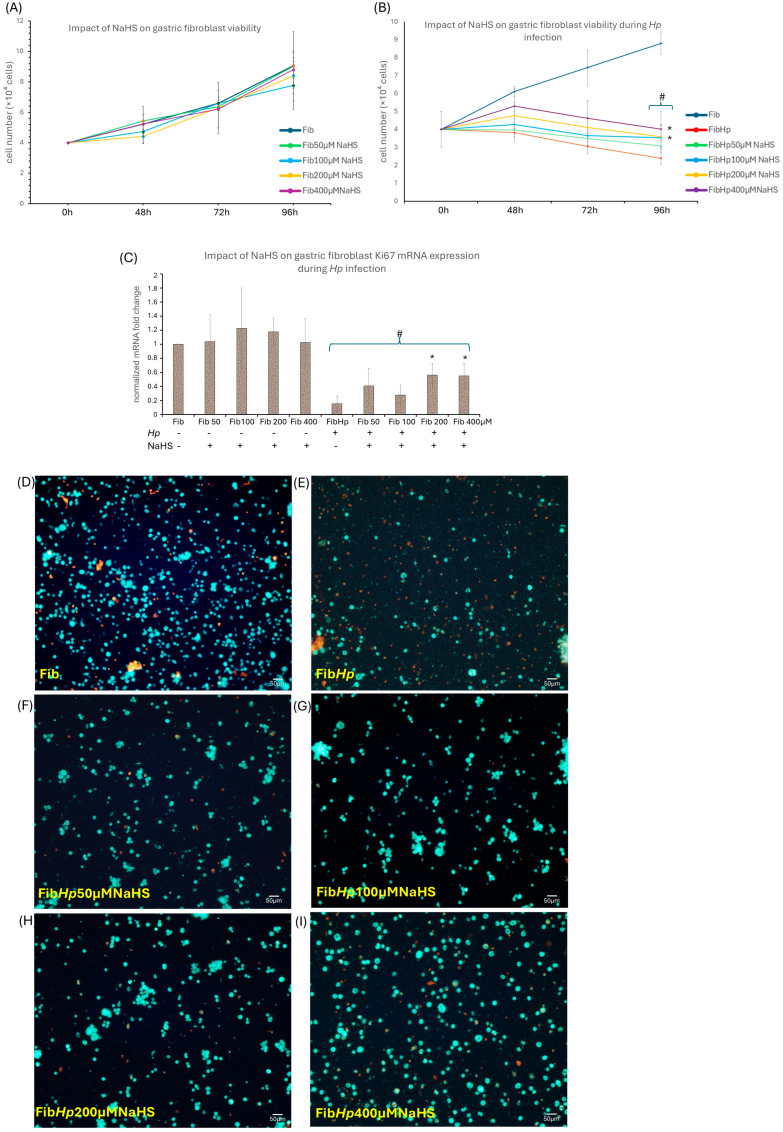
The viability of the control NHGFs, and NHGFs after the addition of NaHS at concentrations of 50–400 µM after 48, 72 and 96 h of culture (**A**). No antibiotics were added. The viability of non-infected and *Hp*-infected NHGFs treated with NaHS (50–400 µM) (**B**). For the viability assay, fibroblasts were seeded at the concentration of 4 × 10^4^ and allowed to grow for 48, 72, and 96 h. Cells were then harvested and counted with automated cell counter. The cell number was additionally verified with the staining with trypan blue and counting in Burker hemocytometer. qPCR analysis of the expression of *Ki67* mRNA. qPCR analyses were normalized to *18S* rRNA and data are presented as fold changes relative to the control group (**C**). Fluorescent microscopy of NHGFs showing strong reduction in viable cell number during Hp infection, with subsequent increase with the increasing dose of NaHS (**D**–**I**). Green: living cells (acridine orange) and red: dead cells (propidium iodide). Results are mean ± SEM of two to six independent experimental repeats. Asterisk (*) indicates a significant change (*p* < 0.05) as compared to the control value (non-infected fibroblasts). Hash (#) indicates a significant change (*p* < 0.05) as compared to *Hp*-infected fibroblasts.

*Hp* infection substantially reduced proliferation and viability of gastric fibroblasts, as demonstrated by markedly lower cell numbers over time (Figure 6B) and a visible loss of viable cells in fluorescent imaging (Figure 5E). The addition of NaHS at 50–400 μM to non-infected fibroblasts did not affect proliferation or viability, as confirmed by total cell counts after 48, 72, and 96 h of experimental procedure (Figure 6A), *Ki67* mRNA levels (Figure 6C) and green/red live-dead staining (Figure 6D). This indicates that NaHS shows no cytotoxic effects within the tested concentration range. In *Hp*-infected fibroblasts, NaHS partially restored proliferation and viability. Cell counts were significantly higher after 96 h for NaHS-treated groups (concentrations from 100 to 400 μM) compared with untreated *Hp*-infected fibroblasts (Figure 6B). Fluorescence images likewise showed a progressive improvement in the number of viable (green) cells in NaHS-treated cultures (Figure 6F–I), consistent with better cell survival. *Ki67* mRNA expression displayed a similar tendency toward higher values at 200 and 400 μM NaHS, although this trend was modest and *Ki67* levels in *Hp*-infected fibroblasts remained below those of non-infected controls (Figure 6C). Taken together, these data indicate that NaHS exerts a partial protective effect on gastric fibroblast growth and survival under *Hp*-induced stress, while remaining non-cytotoxic under basal (non-infected) conditions within the tested concentration range.

### 3.4. The Effect of NaHS Administration on Hp Viability and Its Sensitivity to Metronidazole

**Figure 7:**
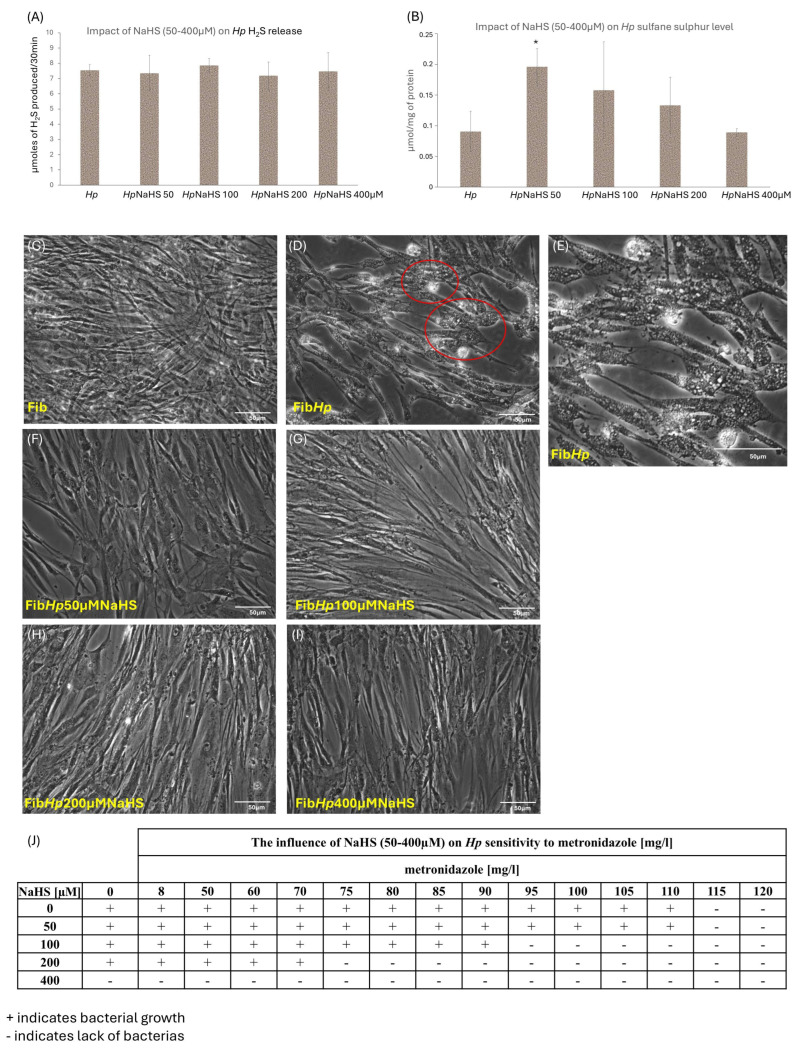
The effects of exogenous NaHS on H_2_S metabolism, adhesion to gastric fibroblasts and metronidazole sensitivity of *Hp*. The impact of NaHS (50, 100, 200 and 400 µM) on *Hp* H_2_S release showing no alterations in H_2_S production levels during 48 h of culture. Experiments were carried out for the three experimental groups after incubation with L-cysteine as a main endogenous substrate for H_2_S producing enzymes (**A**). SS content in *Hp* cultures grown with and without the addition of 50, 100, 200 and 400 µM NaHS. The SS level was expressed as nmoles of SCN produced per 1 mg of protein (**B**). Phase contrast microscopy of NHGFs (**C**), NHGFs infected with *Hp* (**D**,**E**) as well as NHGFs infected with *Hp* with daily administration of 50 µM NaHS (**F**), 100 µM NaHS (**G**), 200 µM NaHS (**H**) and 400 µM NaHS (**I**) showing decreased adhesion of *Hp* to fibroblasts. The influence of NaHS on the sensitivity of Hp to metronidazole presenting increased sensitivity of bacteria after the addition of higher doses of NaHS (200 µM) and high toxicity exerting complete bactericidal effects in the case of 400 µM NaHS (**J**). The data represents mean value from 10 to 15 repeats from three (*) independent experiments ± SEM. Asterisk indicates a significant change (*p* < 0.05) as compared to the control value (*Hp*).

We have shown that *Hp* releases H_2_S at a low, basal level of ~8 µM per 30 min. The administration of NaHS at varying concentrations (50–400 µM) did not induce significant alterations in H_2_S production (Figure 7A). This indicates that bacterial H_2_S synthesis in *Hp* is primarily mediated by endogenous H_2_S-producing enzymes and is largely independent of exogenous NaHS supply, suggesting that under our culture conditions this system operates in a relatively self-regulated, near-maximal range. At a concentration of 50 µM NaHS, an increase in SS level was observed, likely reflecting the utilization of exogenous H_2_S by the bacteria to expand the intracellular pool of reactive sulfane sulfur (Figure 7B). This suggests that with a moderate increase in exogenous H_2_S, *Hp* effectively converts H_2_S into SS, potentially serving as a storage pool or reservoir of redox-active sulfur forms. Notably, Figure 6B quantifies SS directly in *Hp*, whereas Figure 5E reflects SS measured in *Hp*-infected fibroblasts. Thus, the SS increase observed in bacteria at 50 µM NaHS (Figure 7B) may not necessarily translate into a detectable increase in fibroblast SS (Figure 5E), reflecting compartment-specific sulfur handling. *Hp* infection may elevate fibroblast SS via accelerated H_2_S turnover and redistribution of H_2_S-derived sulfur into oxidized and protein-bound pools (a functional plateau at low-dose NaHS), whereas bacteria seem to efficiently channel moderate exogenous H_2_S into intracellular SS at 50 µM [53]. At higher NaHS concentrations (100–400 µM), SS levels, although numerically elevated, did not differ significantly from untreated controls, which may reflect saturation or feedback limitations of sulfur conversion pathways, especially when considering the toxic effect of 400 µM NaHS (Figure 7B). This decline may also relate to inhibitory effects of NaHS on thiol-dependent enzymes, including urease, via cysteine modification, a mechanism described for other fast-releasing H_2_S donors such as sulforaphane and isothiocyanates [49]. Collectively, these observations imply a biphasic response in *Hp*, an optimal exogenous H_2_S concentration (~50 µM) enhances SS formation, whereas higher concentrations trigger regulatory constraints or metabolic disruption and prolonged exposure to 400 µM NaHS becomes cytotoxic (Figure 7J). Phase-contrast imaging confirmed that *Hp* adheres tightly to gastric fibroblasts, forming dense microcolonies (Figure 7D,E) and that NaHS at higher concentrations (200–400 µM) visibly reduces this adhesion (Figure 7F–I). At 400 µM NaHS, this loss of adhesion coincided with a marked reduction in bacterial viability in the MBC assay (Figure 7J), suggesting that high-dose NaHS may hinder bacterial interaction with host receptors and limit virulence factor translocation. To clarify the contribution of direct H_2_S signaling effects in fibroblasts from those secondary to reduced bacterial contact, future studies should quantify the kinetics of *Hp* adhesion loss under increasing NaHS concentrations. To determine whether NaHS interferes with standard antibiotic therapy, we assessed *Hp* sensitivity to metronidazole (doses of 8, 50, 60, 80, 85, 90, 95, 100, 105, 110, 115 and 120 mg/L) (Figure 7J). NaHS did not diminish antibiotic efficacy. Instead, increased bacterial death became evident starting at 100 µM NaHS and progressed in a dose-dependent manner (Figure 7J). It was assessed visually as the reduction in bacteria growth which was further verified by transferring the bacteria suspension from the wells onto Columbia Agar with 5% fresh horse blood. Collectively, these findings indicate that while NaHS does not alter endogenous bacterial H_2_S production, it affects bacterial sulfur metabolism, reduces bacterial adhesion and enhances antibiotic susceptibility at higher concentrations.

## 4. Discussion

We have shown that, in accordance with our previous results [6,7,8], *Hp* infection profoundly reprograms gastric fibroblasts toward a CAF-like phenotype, characterized by increased Twist (mRNA, protein and nuclear localization), upregulation of CAF markers *FAP* and *FSP*, elevated *IL-8* and *HGF* expression which is accompanied by robust activation of NF-κB (IKKα/IKKβ/p65 phosphorylation) and STAT3 signaling. In this study we have used the well-characterized high-virulence *Hp* strain ATCC 43504 (*cagA+vacA+* (*s1*/*m1*)) with the strongest ability to induce Twist expression in fibroblasts. The virulent characteristics of this strain are consistent with its documented pathogenic profile [39]. In contrast, a *cagA− vacA−* (*s2*/*m2*) strain failed to activate gastric fibroblasts, in agreement with our previous findings [40], supporting the rationale for using a high-virulence reference strain. Across experiments, primary gastric fibroblasts were obtained from two to three independent donors. Moreover, *Hp* induced *Twist*, *STAT3*, *IL-6* and *FAP* expression in non-gastric BJ fibroblasts, and NaHS consistently suppressed these markers, indicating that the observed responses are not restricted to a single fibroblast background. Nonetheless, broader validation across additional donor-derived fibroblasts and primary CAFs will be required to fully assess the generalizability of our findings. The robust activation of NF-κB (IKKα/IKKβ/p65 phosphorylation) and STAT3 signaling align with studies linking chronic *Hp* infection to persistent NF-κB activation *via cytotoxin-associated gene A* (*CagA*), delivered through the type IV secretion system (T4SS), and recognition by PRRs including TLRs, growth factor (GF), and cytokine receptors [3,6,9,10,11,54]. We have previously demonstrated increased expression of TLR2 and TLR4 in infected fibroblasts [6]. These receptors, both associated with GC risk, have been shown to enhance CagA-driven inflammation. Mechanistically, bacterial lipoproteins activate TLR2, followed by TLR4 engagement via autocrine and lipopolysaccharide (LPS)-dependent signaling [9]. TLR2 was shown to trigger NF-κB via MyD88/interleukin-1 receptor-associated kinase (IRAK) [10,11], while TLR4 was shown to activate STAT3 in non-gastric tissues [12], indicating parallel NF-κB and STAT3 activation. Our data suggest that similar mechanisms operate in gastric fibroblasts, facilitating the parallel induction of NF-κB and STAT3 and thereby reinforcing previous reports of sustained STAT3 activation by *CagA+* strains [55]. In addition, it has been shown that unphosphorylated CagA promotes Janus kinase (JAK)/STAT3 signaling, whereas its phosphorylated form enhances extracellular signal-regulated kinase/mitogen-activated protein kinase (ERK/MAPK) pathways [9]. NF-κB and STAT3 have been shown to collaboratively regulate genes involved in inflammation, reprogramming, survival and metastasis [28,36,37], facilitating crosstalk between malignant cells and TME [38,55]. In this context, *Hp*-induced fibroblast activation further promotes immune evasion and metastatic progression, progressively reshaping the gastric stroma into a tumor-supportive niche [4,6,7,8]. Consistent with this notion, the heightened secretion of IL-6, IL-8 and HGF by *Hp*-infected fibroblasts underscores the convergence of these pathways in driving a tumor-promoting CAF phenotype. Both NF-κB and STAT3 can be rapidly activated, yet they are regulated by distinct signaling mechanisms. Nonetheless, they share overlapping downstream genes that often require cooperative transcription [10]. Several NF-κB family members, notably RelA/p65 and p50, physically interact with STAT3. Cytokines and GFs upregulated by NF-κB in the TME, including IL-6, often termed the “coupler” of NF-κB/STAT3 signaling [38] or HGF, further drive STAT3 activation in both tumor and stromal cells, a phenomenon also observed in GC [30,55]. Once activated, STAT3 can induce a subset of essential genes for its own positive feedback loop, such as IL-6, EGF, cell surface receptors (e.g., EGFR, c-Met), or proto-oncogenes like K-Ras [36,56,57,58]. Moreover, activated STAT3 has been shown to prolong the nuclear presence of NF-κB, potentially ensuring constitutive NF-κB activity in cancer cells despite elevated levels of the anti-inflammatory cytokine TGF-β [7,38,59]. Consequently, this synergy fosters a self-sustaining loop that sustains inflammatory signals. Our results demonstrate that NaHS markedly counteracts *Hp*-driven fibroblast activation. Across all tested concentrations, NaHS rapidly reduced *Twist*, *STAT3*, *IL-6*, *IL-8*, *FAP* and *HGF* expression within 48 h of infection and prevented their re-induction upon secondary exposure, ultimately restoring transcript levels to those of non-infected fibroblasts by 96 h. This transcriptional normalization was paralleled by suppression of Twist protein accumulation and nuclear translocation, as well as attenuation of NF-κB (p65) and STAT3 phosphorylation, back to control levels. Importantly, a similar protective pattern was observed in non-gastric fibroblasts, suggesting that this effect is not tissue restricted. Importantly, NaHS also conferred partial protection against reinfection, attenuating the secondary rise in *STAT3*, *IL-6*, *IL-8* and *FAP* mRNA expression. Collectively, these data suggest that NaHS blunts *Hp*-induced fibroblast reprogramming and concomitant NF-κB/STAT3 activation, pointing to H_2_S as a potential modulator of stromal inflammation in the gastric niche. The concentrations of NaHS used in this study (50–400 µM) fall within ranges commonly applied to model acute, transient H_2_S exposure in mammalian cells. This is consistent with previous work showing that concentrations ≥50 µM are typically required to generate physiologically relevant peak H_2_S levels in vitro, because NaHS releases H_2_S rapidly and only a small fraction persists in the extracellular milieu for more than a few minutes. Kinetic analyses by Lee et al. showed that 400 μM NaHS generates a rapid H_2_S peak close to the nominal concentration that dissipates within about 1 h, whereas the slow-releasing donor GYY4137 at the same concentration produces only low (<20 µM) but sustained H_2_S levels for up to 7 days [49]. Importantly, that study also demonstrated marked functional differences between these donors. GYY4137 displayed dose-dependent cytotoxicity toward several human cancer cell lines while sparing normal fibroblasts, induced caspase-dependent apoptosis in MCF-7 cells during prolonged exposure, and suppressed tumor growth in xenograft models. In contrast, NaHS was less potent and inactive in several of the tested cancer cell lines. Collectively, these data highlight that the biological effects of H_2_S donors depend strongly on their release kinetics and support the use of micromolar-to-sub-millimolar NaHS concentrations to model short-lived H_2_S bursts in vitro. Consistent with this framework, preliminary assays using the slow-releasing donor GYY4137 (50 and 100 µM) in *Hp*-infected fibroblasts showed reductions in *Twist* and *IL-6* mRNA expression that resembled the effects of NaHS. Although these are preliminary data, the findings suggest that both fast- and slow-releasing H_2_S donors may reduce *Hp*-induced fibroblast activation, likely by acting on shared signaling pathways rather than simply through differences in their H_2_S-release kinetics. These parallel effects of NaHS and GYY4137 support the interpretation that the fibroblast responses observed in our system arise from H_2_S itself rather than from donor-specific physicochemical properties. By demonstrating convergent suppression of *Hp*-induced *Twist* and *IL-6* expression with two mechanistically distinct H_2_S donors, our data strengthen the conclusion that H_2_S may act as the critical mediator underlying these effects. Further studies comparing donors with different H_2_S-release rates will be needed to determine whether prolonged, low-level H_2_S delivery provides the same, stronger, or a distinct form of protection compared with a short, high-amplitude H_2_S burst. Our findings correspond with studies showing that H_2_S prevents IκB degradation, blocking NF-κB translocation and proinflammatory cytokine production [30], and modulates redox-dependent IKK-β modifications, promoting an anti-inflammatory state [60]. Such NF-κB/STAT3 inhibition in fibroblasts is expected to dampen broader inflammatory cascades (JAK/STAT3, MAPK, TLR/NLR) and to favor the expression of genes involved in sulfur metabolism, heat-shock responses and DNA repair [30,33,34,35,60,61]. Collectively, these findings support the role of H_2_S in disrupting the NF-κB/STAT3 loop accompanied by the suppression of gastric fibroblast activation and their pro-tumorigenic transformation during Hp infection. Although not directly assessed here, H_2_S has been additionally reported to enhance IL-10 and antioxidant responses via a nuclear-factor-E2-related factor-2 (Nrf2)-dependent pathway, in which H_2_S-induced S-sulfhydration of Keap-1 enables Nrf2 nuclear translocation [33,34], represses *IL-6* and *IL-1β* expression, induces antioxidant proteins (HO-1, Trx, GST, GPx, TrxR) and reduces ROS [17,33,34,35]. Thus, the activation of Nrf2 may provide an additional mechanism that reinforces the anti-NF-κB/STAT3 activity of H_2_S in the gastric microenvironment. The change in medium osmolarity and Na^+^ concentration induced by the highest NaHS dose is only approximately a fraction of a percent of their baseline values, so it is far below the range typically required to affect mammalian cells. This interpretation is consistent with experimental data from other cell types. In bovine aortic endothelial cells, exposure to clearly hyperosmolar medium (~460 mOsm/L, more than 100 mOsm above physiological osmolarity) did not reduce viability, and in a separate study an increase in medium osmolarity from ~330 to ~400 mOsm improved chondrocyte survival after photoencapsulation compared with hypoosmotic conditions [50,51]. Similarly, in NPMSCs, experimental osmotic manipulations in the range of about 100–300 mOsm are used to induce measurable changes in proliferation, apoptosis, senescence and ECM metabolism [52]. ATCC guidelines indicate that most vertebrate cell lines are maintained within 260–320 mOsm/kg and can tolerate a relatively broad osmolarity range. Classic studies on hyperosmotic stress further show that osmosensitive pathways, such as MAPK cascades, NFAT5/TonEBP and heat-shock responses, are typically activated only when extracellular osmolality is increased by several dozen mOsm above physiological levels, and that fibroblasts and other cell types can compensate moderate osmotic challenges of about 50–100 mOsm through volume-regulatory mechanisms without major changes in viability or basal signaling [62,63]. In parallel, work on ion-dependent volume regulation shows that only changes in extracellular Na^+^ concentration of several millimolar are sufficient to elicit robust signaling responses [64,65]. Thus, a shift in osmolarity of less than 1 mOsm and minimal Na^+^ change produced by 400 µM NaHS in our system are at least two orders of magnitude below the thresholds typically required to trigger hyperosmotic stress pathways, making nonspecific osmotic or Na^+^-dependent mechanisms an unlikely explanation for the observed NaHS effects. These assumptions were confirmed by control experiments in which we treated fibroblasts with 50 and 400 µM NaCl for 96 h. The fibroblasts showed no significant changes in *Twist* or *IL-6* mRNA expression (Supplementary Data). We have selected Twist and IL-6 because they are robust and highly sensitive markers of *Hp*-induced fibroblast activation in our model. Twist is a central regulator of CAF-like reprogramming and in our experiments is consistently upregulated in *Hp*-exposed fibroblasts [4,6,7,8], in parallel with NF-κB/STAT3 activation. IL-6 is a key component of the NF-κB/IL-6/STAT3 axis and one of the earliest and most pronounced cytokine responses to *Hp* [6]. If NaCl had any nonspecific osmotic, ionic or stress-related effects, we would expect to detect changes in the expression of these genes. Moreover, NaHS alone did not induce statistically significant changes in the mRNA expression of CAF-associated proinflammatory and tumor-promoting markers, which indicates that the effects observed in *Hp*-infected fibroblasts reflect modulation of infection-induced signaling rather than nonspecific osmotic changes. Our results further revealed that *Hp* infection profoundly remodeled fibroblast sulfur metabolism. AzMC fluorescence quantification revealed a reduction in free H_2_S in infected cells, implying accelerated turnover and redirection of H_2_S into oxidized or protein-bound sulfur pools. At the molecular level, *Hp* induced CBS and MPST expression and activity and upregulated TST, enzymes central to H_2_S biosynthesis and oxidation, while simultaneously reducing measurable H_2_S release and elevating persulfide (SS) levels. Together, these features point to an adaptive, stress-protective remodeling of sulfur metabolism, in which intensified H_2_S turnover and persulfide accumulation support redox buffering and contribute to fibroblast reprogramming toward a CAF-like phenotype. In contrast, exposure to exogenous H_2_S (NaHS, 50–400 μM) did not substantially alter *CBS*, *MPST*, *CSE* or *TST* expression in non-infected fibroblasts and produced only modest changes in MPST activity and SS levels, suggesting that NaHS does not disrupt basal H_2_S metabolism but rather acts downstream of its enzymatic regulation. The lack of a significant change in enzyme levels and activity could mean that the system is maintaining homeostasis, and the observed small increase in SS represents a physiological response rather than a pathological alteration. These changes reflect a coordinated response to oxidative stress, aligning with known mechanisms where *Hp* virulence factors such as CagA and LPS induce NF-κB activation and ROS, both of which can upregulate CBS via inflammatory and hypoxia-responsive pathways [6,26,36,66,67]. CBS, a key H_2_S-producing enzyme, has been shown to contribute to redox defense by supporting glutathione (GSH) synthesis [18,30,60,68]. Its depletion is known to increase oxidative stress and suppress NF-κB, while overexpression, although context-dependent, often correlates with tumor progression and poor clinical outcomes, as observed in GC samples and TCGA analyses [18]. Although CBS is primarily a cytosolic enzyme, it may redistribute to mitochondria (via Lon proteases) or translocate to the nucleus (via SUMOylation) under conditions such as hypoxia or ischemia [18,68,69]. In cancer cells, this redistribution gains functional relevance, as mitochondrial CBS has been shown to support cell survival by enhancing energy metabolism [70]. Consistently, CBS overexpression leads to broad transcriptional reprogramming, altering expression of more than 350 genes linked to glycolysis, hypoxia response, EMT, proliferation, migration, invasion, ECM remodeling, angiogenesis, multidrug resistance, and signaling pathways including NF-κB, STAT3, KRAS, p53, Wnt, and EMT [18,70,71]. Importantly, the functional outcomes of CBS overexpression are context dependent. In human colorectal adenocarcinoma HT-29 cells, which naturally express high CBS levels, further CBS elevation reduced viability, proliferation, tumor growth, and metastasis, demonstrating that excessive CBS activity can under some circumstances be detrimental to cancer cell growth. Conversely, in cells with low baseline CBS expression, its overexpression promotes tumor progression [18,72]. This paradox aligns with the biphasic, bell-shaped nature of H_2_S, where optimal levels support cell survival, but excessive H_2_S suppresses cell metabolism, impairs proliferation and induces cytotoxicity. Together, these observations highlight that CBS activity exerts bidirectional effects on tumor biology, where both inadequate and excessive CBS levels can disrupt tumor cell survival and function. Consistent with this context-dependent role, silencing CBS in cancer models has been shown to reduce oxygen consumption, ATP production and mitochondrial integrity, while increasing oxidative stress (via reduced glutathione and elevated ROS) and suppressing NF-κB activation [18,73]. Moreover, in vivo CBS silencing has been shown to impair ovarian cancer growth and angiogenesis and to sensitize tumors to cisplatin, underscoring its potential as a therapeutic target [18,74]. In the context of *Hp* infection, moderate CBS upregulation seems to help to preserve redox homeostasis, mitigate ROS-mediated damage, assisting with the reprogramming of fibroblasts into tumor-supportive CAFs. It has been previously shown that CBS induction is driven by inflammatory signaling through NF-κB and STAT3 and may also be influenced by hypoxia-associated pathways such as HIF-1α activation [6,18]. This connection raises the possibility that *Hp*-driven NF-κB/STAT3 activation not only reprograms fibroblasts phenotypically but also influences CBS expression as part of a metabolic adaptation to inflammation-induced stress. Furthermore, other H_2_S-generating enzymes such as CSE and MPST have also been postulated to contribute to the tumor-supportive metabolic environment. While their roles vary by cancer type, they often act additively or cooperatively with CBS to sustain cellular proliferation, bioenergetics, and stress resistance [18,73]. MPST plays a multifaceted role beyond H_2_S production, being a key source of polysulfides (H_2_Sn) and reactive sulfur species (RSS) with strong regulatory functions in cell signaling. H_2_Sn, the main chemical form of the SS pool, can modify protein thiols via S-sulfhydration (persulfidation), altering protein conformation and activity, thereby facilitating redox signaling, scavenging ROS, and maintaining redox homeostasis [18,19]. Through these actions, H_2_Sn influence critical cellular processes such as apoptosis, inflammation, cell proliferation, and mitochondrial bioenergetics and integrity. In the tumor context, H_2_Sn derived from MPST support angiogenesis, oxidative stress resistance, and metastatic traits, while also shaping immune responses and cytokine networks, thereby promoting TME remodeling [18,19]. In tumors such as human colon cancer, MPST expression is frequent and correlates with poorer survival outcomes, similarly to CBS [18,70,75]. MPST upregulation has been observed in tumor cells with multidrug resistance or stem-like phenotypes, particularly during recovery from cytotoxic stress, suggesting a role in therapy adaptation [18,76]. Functionally, MPST mirrors CBS in sustaining tumor-promoting processes including EMT, angiogenesis and bioenergetic stability, thus reinforcing its importance in preserving the malignant potential of the TME [18,19]. Notably, the impact of H_2_S on *MPST* expression is highly context dependent. Identical H_2_S concentrations can either upregulate or deplete MPST depending on the intrinsic sulfide tolerance of the cell type [20,25,77]. Importantly, *MPST* expression is also controlled by NF-κB and sensitive to oxygen availability, highlighting its upregulation under hypoxia and inflammation [20]. *MPST* shares a bidirectional promoter with *TST*, implying coordinated regulation of sulfur metabolism at the transcriptional level [20]. Thus, MPST emerges not only as a redox modulator but also as a contributor to tumor resilience and progression, particularly under inflammatory and hypoxic stress. TST, traditionally linked to cyanide detoxification, also emerged as a stress-responsive enzyme. Increasing evidence suggests that TST participates in a variety of additional cellular processes, including the transport of sulfur and selenium in bioavailable forms [21], mitochondrial import of *5S* rRNA [78], and the restoration of iron-sulfur (Fe-S) clusters in key mitochondrial proteins such as aconitase and respiratory chain complexes [21]. TST supports also ROS detoxification by generating RSS through thiosulfate conversion, contributing to redox signaling and cellular function, as shown in models of inflammation and cancer [20,21,25,76]. In the colon, it serves as a major H_2_S-detoxifying enzyme, particularly during inflammatory conditions such as ulcerative colitis [25]. TST expression is context dependent, often downregulated in several cancers (e.g., colorectal tissue and hepatocellular carcinoma) [16,17,21,76], and its reduced activity is also seen in some lines such as 4T1 mammary tumor cells, suggesting altered sulfur metabolism. By contrast, increases in TST are reported mainly under specific conditions, e.g., during differentiation of colorectal models (HT-29) and in isogenic colonic epithelial organoids, or under inflammatory/oxidative cues [25,79,80,81]. Thus, variation in TST reflects cell state and microenvironment rather than a uniform hallmark of a given tumor type. Additional evidence of TST involvement in stress adaptation comes from other systems. In the SAMP8 mouse model of Alzheimer’s disease, increased *TST* expression was observed in response to cognitive decline and elevated levels of pro-inflammatory cytokines (IL-1β, TNF-α, IL-6) [82]. Similarly, in 3T3-L1 adipocytes, TST upregulation by sodium thiosulfate (STS) or diallyl disulfide (DADS) reduced cytokine secretion and ROS production during palmitate-induced inflammation [83]. Conversely, TST deficiency has been shown to disrupt the balance of ROS and RSS, impair mitochondrial oxidative phosphorylation, and alter NRF2-Keap1 signaling in the cortex of TST-/-mice, leading to exacerbated oxidative stress and diminished antioxidant defense [84]. Given *Hp*-induced NF-κB activation and the reported sensitivity of sulfur-metabolizing enzymes to inflammatory/hypoxic cues [6,20,21,25,83,84], the TST increase we observe likely mirrors NF-κB-linked metabolic remodeling; however, direct NF-κB binding to the *TST* promoter has not been demonstrated in our study. Together with MPST, TST acts as a central regulator of SS homeostasis, keeping polysulfide levels within the range that supports physiological redox signaling but avoids harmful accumulation [16,18,21]. Consistent with these findings, our experimental model demonstrated that *Hp* infection enhances H_2_S metabolism, as evidenced by decreased free H_2_S levels in supernatants and increased SS compound concentrations. These changes suggest a shift toward intensified H_2_S oxidation, particularly via TST-mediated sulfur transfer, as a mechanism to prevent toxic H_2_S and ROS accumulation [70,83,84]. Thus, TST upregulation appears to be a stress-adaptive mechanism aimed at preserving redox balance, mitigating oxidative injury, and modulating inflammation, potentially under the influence of NF-κB signaling. Whether NF-κB directly regulates *TST* transcription or does so indirectly through broader sulfur metabolism and oxidative stress pathways remains to be clarified. Overall, our data, together with prior reports, indicate that coordinated upregulation of CBS, MPST, and TST under NF-κB/STAT3 signaling may contribute to fibroblast survival and reprogramming toward a CAF-like state, thereby supporting tumor-promoting functions such as ECM remodeling, angiogenesis, and immune modulation [4,8,16,17,18,19] in the *Hp*-altered microenvironment. Beyond its effects on fibroblast activation, NaHS also influenced the *Hp*-driven remodeling of sulfur metabolism. NaHS attenuated the upregulation of H_2_S-related enzymes triggered by *Hp*. Our data show that NaHS modulates the sulfur metabolic response induced by *Hp*. In infected fibroblasts, NaHS reduced CBS and MPST mRNA and protein expression, and lowered *TST* mRNA levels. Although *MPST* mRNA remained elevated after reinfection, and residual TST activity persisted above baseline, suggesting that fibroblasts may preserve a degree of TST-mediated sulfur transfer as part of a stress-adaptive redox response, the overall enzyme expression and activity shifted toward levels closer to those of non-infected fibroblasts. NaHS did not markedly alter SS content at lower concentrations, whereas higher doses reduced SS levels, indicating that exogenous H_2_S decreases endogenous H_2_S enzyme induction without promoting further persulfide accumulation. Collectively, these data indicate that NaHS reshapes infection-induced sulfur metabolism attenuating endogenous H_2_S-producing enzyme induction without amplifying persulfide storage. This suggests the presence of feedback regulation within the H_2_S network, whereby exogenous H_2_S tempers excessive enzyme upregulation rather than simply adding to the metabolic pool. Overall, these findings indicate that NaHS counteracts *Hp*-induced remodeling of H_2_S-generating and H_2_S-oxidizing pathways, dampening metabolic changes associated with fibroblast activation. These metabolic effects of NaHS may be functionally relevant in the context of fibroblast activation. By attenuating CBS, MPST and TST induction without increasing persulfide storage, exogenous H_2_S likely prevents the infection-induced shift toward intensified sulfur turnover and RSS accumulation that accompanies the CAF-like transition. This pattern is consistent with studies showing that exogenous H_2_S donors stabilize redox homeostasis and limit inflammatory signaling rather than amplifying sulfur metabolic activity [30,33,60]. Such feedback regulation may protect fibroblasts from entering a high-RSS, high-oxidative-stress state that favors pro-tumorigenic signaling, including NF-κB/STAT3 activation, metabolic reprogramming and cytokine release [18,19,21]. Importantly, the ability of NaHS to moderate *Hp*-induced sulfur metabolic remodeling complements its inhibitory effects on fibroblast activation markers and inflammatory pathways. The convergence of these actions is consistent with a model in which H_2_S modulates NF-κB/STAT3 signaling and helps to stabilize redox homeostasis, while constraining sulfur-based redox signals that would otherwise sustain the activated fibroblast phenotype. The combined effects of NaHS on signaling, metabolism and activation of *Hp*-infected fibroblasts prompted us to examine how *Hp* and H_2_S influence their proliferation and viability. We have shown that *Hp* infection substantially reduced both parameters, consistent with previous reports indicating that *Hp* profoundly affects fibroblast growth and survival through multiple mechanisms, ultimately reducing cell numbers [7,8]. This aligns with prior evidence that *Hp* impairs cell viability and proliferation through a combination of oxidative stress, disruption of cell cycle regulation, inflammation and the action of specific virulence factors [85,86]. In fibroblasts, these processes may contribute to apoptosis or growth arrest, although not all of them have been directly documented in our model. *Hp* has been shown to induce apoptosis, particularly in gastric epithelial cells, mainly via virulence factors that directly activate the intrinsic apoptotic pathway [3,6,9,10,11,12,54,85,86,87]. In addition, *Hp* has been shown to increase the sensitivity of cells to death receptor-mediated apoptosis (extrinsic pathway) in a manner independent of VacA and CagA, indicating that host immune factors, alongside bacterial determinants, are important contributors to gastric mucosal damage during infection [85,86,87]. VacA can induce concentration- and time-dependent cell death via several mechanisms, including membrane channel formation, vacuolization, mitochondrial dysfunction and autophagy [85,86,87]. CagA, in turn, drives NADPH-oxidase-dependent ROS production and modulates NF-κB/STAT3 signaling, thereby integrating stress and inflammatory cues and facilitating both pro-apoptotic and pro-survival transcriptional programs [6,9,10,11,12,54]. Additional factors such as OipA, LPS, gamma-glutamyl transpeptidase (GGT) and glycolic acid extract (GE) further enhance metabolic and oxidative stress and thereby potentiate the activation of intrinsic and in some contexts, extrinsic apoptotic cascades [85,86,87]. *Hp*-induced ROS function as upstream integrators of stress signaling, with their consequences depending on intensity and duration [88,89,90]. It has been shown that acute, high levels of ROS promote apoptotic cell death, whereas chronic, sublethal ROS sustain activation of signaling networks, including NF-κB, STAT3 and HIF-1α, which drive fibroblast activation and microenvironmental remodeling [3,6,9,10,11,12,54,85,86,87]. In fibroblasts, it has been shown that ROS-induced NF-κB and HIF-1α activation upregulates ECM genes and myofibroblast markers [91], while NF-κB/STAT3 signaling delays apoptosis under moderate stress but, when damage exceeds compensatory capacity, can also promote its execution [87,92]. *Hp*-induced cytokines such as IL-1β, IL-6 and IL-8 further amplify ROS and stress through NF-κB/STAT3-dependent loops [4,6,7,8,87], reinforcing impairment of cell survival and proliferation. STAT3, known to support tumor growth via mitochondrial gene expression and metabolic reprogramming (Warburg effect), has been shown to cooperate with NF-κB to propagate mitochondrial dysfunction and to reduce apoptosis when chronically overactivated [93]. Thus, NF-κB/STAT3 form a context-dependent regulatory hub that integrates ROS with cell-fate decisions, balancing adaptation and fibroblast remodeling versus stress-induced death. Interestingly, in parallel, it has been shown that *Hp* disrupts cell cycle regulators suppressing Cyclin D1, cyclin-dependent kinases (CDKs) and mitotic genes, thus halting cell cycle progression in immune cells [94]. This contributes to a shift toward senescence, marked by irreversible growth arrest, senescence-associated secretory phenotype (SASP), ROS elevation and mitochondrial activation [27]. ROS have been shown to further promote senescence by inducing CBS expression, which helps to maintain the non-proliferative state. At the same time, increased H_2_S turnover *via* CBS, CSE and TST contributes to limiting oxidative stress [27,43]. Interestingly, senescence and CAF transformation share overlapping triggers such as TGF-β, TLRs, ROS, hypoxia and pathways like NF-κB, STAT3 and MAPK [4,7,8,27,95,96]. Both states contribute to tumor progression. CAFs remodel and stiffen the ECM, sustain chronic inflammation through secretion of IL-6, IL-8, CXCLs and other chemokines, and release growth factors that promote cancer cell proliferation, invasion, angiogenesis, immune evasion and therapy resistance, collectively organizing a tumor-supportive stroma [4,7,8,95,96]. Senescent cells, through sustained SASP secretion, also remodel the ECM, amplify inflammatory signaling, attract and reprogram immune cells and create permissive niches that enhance tumor growth, invasion and therapy resistance. Their secretome, rich in IL-6, IL-8, TGF-β, MMPs and chemokines, can reinforce CAF-like phenotypes in neighboring fibroblasts and propagate chronic, non-resolving inflammation within the tissue. In this way, senescent cells act not merely as growth-arrested bystanders, but as active architects of a tumor-supportive microenvironment, functionally overlapping with and potentiating CAF-driven processes [27,95]. Thus, it seems plausible that the reciprocal interactions between CAFs and senescent cells exist during tumorigenic tissue transformation. The senescent cells can contribute to CAF induction via the SASP, which includes factors like IL-6, IL-8, and TGF-β that can activate fibroblasts. Conversely, CAFs can influence neighboring cells and potentially induce or maintain a senescent state through paracrine signaling, suggesting a bidirectional relationship where senescence can drive CAF activation and CAFs can help to sustain senescence through paracrine signaling. Understanding these processes could clarify the pathological changes in the gastric stroma during *Hp*-related diseases, including GC, where fibroblasts may potentially follow different but interconnected reprogramming towards senescence and state of activation, each path connected with ROS, H_2_S metabolism, and chronic inflammation. It is also worth considering whether these processes may overlap as stages of cellular activity. Upon *Hp* exposure, a substantial proportion of fibroblasts is eliminated, while the surviving subpopulation becomes activated. Although we did not delineate the death pathways mechanistically, our data show that Hp infection reduces fibroblast proliferation and viability, while the surviving cells engage adaptive NF-κB/STAT3 signaling, and acquire CAF-like features [4,5,6,7,8]. In line with previous studies implicating *Hp*-induced ROS, virulence factors and inflammatory signaling in cell damage [3,6,9,10,11,12,54,85,86,87], our findings support the view that *Hp*-driven loss of fibroblast viability and proliferation arise from multiple interconnected processes, including oxidative stress, cell cycle disturbance, inflammatory signaling and virulence-factor-mediated injury. NaHS alone (50–400 μM) did not affect proliferation or viability, indicating a lack of cytotoxicity in non-infected fibroblasts. In *Hp*-infected cells, NaHS partially counteracted this growth suppression, with higher concentrations generally associated with increased cell counts and viable cell signals and modestly improving *Ki67* expression, although not fully restoring proliferation to control levels. Together, these findings indicate that NaHS provides cytoprotective support under *Hp*-induced stress while remaining neutral under basal conditions, suggesting that H_2_S supplementation could mitigate some of the detrimental cellular consequences of *Hp* infection without negatively affecting healthy fibroblasts. Previous studies have shown that H_2_S not only neutralizes ROS, but also strengthens antioxidant systems such as GSH, SOD and catalase. Mitochondrial H_2_S generated by MPST additionally helps to reduce oxidative stress and by converting cystine to cysteine, supports GSH synthesis [16,17,18,26,30,33,34,35,61,84]. While prior reports indicate that *Hp* induces mitochondrial impairment [85,86,87], H_2_S has been shown to preserve mitochondrial integrity by stabilizing the mitochondrial membrane potential, limiting cytochrome c release and shifting the Bcl-2/Bax ratio towards cell survival [96]. At low, near-physiological concentrations, H_2_S can also support mitochondrial bioenergetics by supplying electrons to the electron transport chain via sulfide:quinone oxidoreductase (SQR), thereby enhancing ATP production and preventing sulfide accumulation that would otherwise inhibit complex IV [97,98]. In addition, H_2_S can improve mitochondrial protein function through S-sulfhydration of targets such as ATP synthase and SIRT3, which promotes stress resilience and limits apoptosis [99]. Notably, the NaHS doses used in our study (50–400 μM) exceed this low physiological range; nevertheless, the cytoprotective trends we observe are consistent with reports that exogenous H_2_S donors can mitigate redox and mitochondrial stress via several, partly overlapping mechanisms, with the most pronounced effect in our model seen at 400 μM NaHS [16,17,18,26,30,33,34,35]. These redox- and mitochondria-protective effects of H_2_S may contribute to the improvement in cell counts and the upward trend in *Ki-67* observed in NaHS-treated *Hp*-infected fibroblasts. Given that oxidative and mitochondrial stress are known to regulate cell cycle checkpoints, and *Hp* has been shown to arrest cells at G_1_/S or G_2_/M by increasing p27/Kip1 and reducing Cyclin D1/Cdk activity [94], it is possible that improved redox/mitochondrial status also alleviates stress-dependent cell-cycle inhibition, although this was not directly assessed in our study. Consistent with this notion, H_2_S has been reported to stimulate cell-cycle-related proteins and promote proliferation [100]. Beyond its redox- and mitochondria-protective functions, H_2_S has also been shown to exert potent anti-inflammatory and pro-survival effects [101] that may further support fibroblast resilience under *Hp*-induced stress. H_2_S inhibits NF-κB activation through IκB stabilization and redox-dependent modulation of IKK-β [60] and additionally suppresses p38 MAPK, JAK/STAT3 and PRR signaling, while upregulating genes involved in sulfur metabolism, HSPs and DNA repair [17,30,35,60,61,84]. Moreover, H_2_S can influence apoptotic pathways (e.g., *via* c-Jun and caspase-3) [61], promote DNA repair through ERK1/2-dependent PARP-1 activation [85], and enhance survival through PI3K/AKT signaling [102]. It also boosts IL-10 production and activates Nrf2 by Keap1 sulfhydration, enabling increased expression of antioxidant enzymes such as HO-1, Trx, GST, GPx and TrxR [33,34,35]. Taken together, our data in agreement with the literature point to a protective, modulatory role of exogenous H_2_S in *Hp*-exposed fibroblasts, but the precise dose window, mitochondrial contributions, and pathway dependencies remain to be clarified in dedicated mechanistic and in vivo studies. Because NaHS was applied in the context of *Hp* infection, it was also important to define how exogenous H_2_S influences the bacterium itself. Our results indicate that *Hp* maintains a low but stable level of endogenous H_2_S production, which was not altered by NaHS, suggesting that bacterial H_2_S synthesis operates in a largely self-regulated range and is not readily increased by exogenous sulfide. However, *Hp* responded to 50 µM NaHS with a rise in SS content, consistent with conversion of exogenous H_2_S into intracellular SS pools. Higher NaHS concentrations did not further elevate SS levels, implying saturation or feedback restriction within bacterial sulfur-conversion pathways and prolonged exposure to 400 µM became cytotoxic. This biphasic pattern aligns with prior observations that modest exogenous H_2_S can expand reactive sulfur pools, whereas higher concentrations impair thiol-dependent bacterial enzymes and metabolic stability [49]. Additionally, NaHS visibly decreased *Hp* adhesion to gastric fibroblasts at higher doses (200–400 µM). At the highest concentration, this reduction coincided with diminished bacterial viability, suggesting that impaired adhesion may reflect both weakened host–pathogen interactions and direct bacterial toxicity. To ensure NaHS does not impair anti-*Hp* therapies, we tested its effect on metronidazole efficacy. NaHS did not compromise metronidazole efficacy. Instead, it enhanced bacterial death at concentrations equal to or higher than 100 µM, with growth inhibition verified by subculture on Columbia Agar. These observations indicate that exogenous H_2_S can enhance antibiotic susceptibility rather than interfere with treatment.

## 5. Conclusions

We have shown that *Hp*-induced inflammation is accompanied by reduced proliferation and viability of gastric fibroblasts. In parallel, *Hp* reprograms these cells toward a CAF-like phenotype, characterized by Twist upregulation and nuclear localization, increased *FAP*/*FSP* expression as well as enhanced secretion of IL-6, IL-8 and HGF. These phenotypic changes occur together with activation of the NF-κB/STAT3 axis and induction of the sulfur-metabolizing enzymes CBS, MPST and TST. Within this experimental context, our data indicate that exogenous H_2_S (administered as the fast-releasing donor NaHS) mitigates *Hp*-induced fibroblast changes, accompanied by attenuation of NF-κB/STAT3 activation and modulation of sulfur-metabolizing enzymes, under conditions of acute, relatively high sulfide exposure. In *Hp*-infected fibroblasts, acute bolus exposure to the fast-releasing donor NaHS (50–400 μM) resulted in reduced *CBS* and *MPST* expression and partial preservation of TST activity. This metabolic shift coincided with lower levels of CAF-associated markers (*Twist*, *FAP*, *FSP*) and decreased *IL-6*, *IL-8*, *HGF* expression. Functionally, under these acute H_2_S conditions, NaHS alone was not cytotoxic for non-infected fibroblasts, whereas in *Hp*-infected cells it partially restored proliferation and viability. Together, these observations indicate that short, high-amplitude H_2_S exposure can counteract several components of the *Hp*-induced fibroblast response in our in vitro model. Importantly, these effects cannot be attributed to nonspecific osmotic or Na^+^- related alterations. The NaHS concentrations used in this study (50–400 μM) fall within ranges commonly applied to model acute, transient H_2_S exposure, and preliminary observations with the slow-releasing donor GYY4137 suggest that prolonged, low-level H_2_S delivery may similarly reduce *Hp*-induced fibroblast activation. These exploratory results also support the interpretation that the observed fibroblast responses are driven by H_2_S itself rather than by donor-specific properties. Further comparative studies will be needed to determine whether sustained H_2_S exposure provides protection equivalent, stronger, or qualitatively distinct from short, high-amplitude H_2_S bursts. The highest NaHS dose changed medium osmolarity and Na^+^ concentration only minimally, far below levels known to affect mammalian cells. This was supported by additional control NaCl experiments showing no changes in *Twist* or *IL-6* expression and by the lack of NaHS effects on CAF-associated markers in non-infected fibroblasts. Thus, the NaHS effects observed in *Hp*-infected fibroblasts reflect modulation of infection-induced signaling rather than physicochemical artifacts. We have also directly examined how exogenous H_2_S affects *Hp*. Under our conditions, *Hp* maintained a low, stable level of endogenous H_2_S that was not altered by NaHS, indicating that its H_2_S synthesis is largely self-regulated and not easily increased by exogenous sulfide. At 50 μM NaHS, SS content rose, consistent with conversion of exogenous H_2_S into SS pools, whereas higher NaHS concentrations did not further elevate SS and prolonged exposure to 400 μM was cytotoxic. At 200–400 μM, NaHS visibly reduced *Hp* adhesion to gastric fibroblasts, and at 400 μM no viable bacteria were detected. Importantly, NaHS did not compromise metronidazole efficacy. Instead, it enhanced bacterial death at concentrations ≥ 100 µM. These observations indicate that exogenous H_2_S can enhance antibiotic susceptibility rather than interfere with treatment. According to previous studies in other inflammatory and oxidative-stress models, H_2_S may attenuate *Hp*-induced fibroblast activation by inhibiting major signaling pathways that are also triggered during *Hp* infection, including NF-κB/STAT3 signaling. In addition, H_2_S may activate Nrf2 through Keap1 sulfhydration, thereby enhancing antioxidant responses and lowering ROS, which would otherwise potentiate NF-κB/STAT3 activity. By tempering *Hp*-induced CBS, MPST and TST upregulation and preventing excessive accumulation of RSS, exogenous H_2_S may also influence the metabolic remodeling associated with fibroblast activation. The improved fibroblast viability observed at lower NaHS doses under *Hp*-induced stress may represent a functional consequence of the signaling and metabolic effects outlined above. According to the literature, H_2_S may support cell survival by reinforcing antioxidant defenses, preserving mitochondrial function, and modulating cell-death and cell-cycle regulators. Taken together, these literature-based mechanisms provide a plausible explanation for the NaHS-mediated protection of *Hp*-exposed fibroblasts in our model. Based on our data, modulation of H_2_S signaling may be considered as a possible approach to attenuate *Hp*-dependent fibroblast activation and to help maintain fibroblast homeostasis, although its relevance in physiological or in vivo settings remains to be determined. Notably, no antibiotics were included during all assays; therefore, the observed rescue reflects a direct effect of NaHS on fibroblasts at lower H_2_S doses. To clarify the extent to which our observations reflect direct H_2_S signaling in fibroblasts at higher H_2_S doses versus reduced bacterial contact, future studies should quantify the kinetics of *Hp* adhesion loss across increasing NaHS concentrations. The overall pattern supports a dose-stratified model in which low-to-moderate NaHS primarily exerts cytoprotective signaling, whereas higher bolus exposure may additionally limit host–pathogen interaction. The impact of H_2_S on fibroblast reprogramming toward a CAF phenotype warrants further investigation using slow-releasing H_2_S donors (e.g., GYY4137 or newer constructs), which provide more stable, low-level exposure closer to endogenous signaling and reduce artifacts associated with rapid H_2_S release. Assessing such donors alongside NaHS would help clarify their relative ability to restrain *Hp*-induced fibroblast activation and preserve cell survival, while complementary pathway-targeted approaches will be required to define the contribution of NF-κB/STAT3 inhibition versus Nrf2 activation and sulfur-metabolic remodeling. Our in vitro observations are hypothesis-generating. As a limitation, these conclusions are based on a single high-virulence *Hp* strain and a limited number of fibroblast donors; therefore, broader studies including additional donor-derived fibroblasts and other virulent *Hp* strains will be required to confirm the generalizability of our findings. It will also be important to explore whether H_2_S affects already existing CAFs, including the possibility that it may modulate their inflammatory or tumor-promoting functions. Additionally, whether similar fibroblast-preserving and anti-CAF effects can be reproduced in vivo and ultimately hold translational relevance for modifying the pro-inflammatory gastric microenvironment during *Hp* infection remains an important question for future studies.

## Figures and Tables

**Table 1:** qPCR primer list.

Gene	Forward Primer Sequence	Reverse Primer Sequence
*18S* ^1^	5′-CGATCCAATCGGTAGTAGCG-3′	5′-GTAACCCGTTGAACCCCATT-3′
*IL6*	5′-TTCTGCCAGTGCCTCTTTGCTG-3′	5′-AGACAGCCACTCACCTCTTCAG-3′
*STAT3*	5′-CTTTGAGACCGAGGTGTATCACC-3′	5′-GGTCAGCATGTTGTACCACAGG-3′
*IL8*	5′-GAGAGTGATTGAGAGTGGACCAC-3′	5′-CACAACCCTCTGCACCCAGTTT-3′
*FAP*	5′-AGCCATATGGGGATGGTCCT-3′	5′-TGTTGGGAGGCCCATGAATC-3′
*HGF*	5′-CTCATCTCCTCTTCCGTGGACA-3′	5′-GAGAGTTGGGTTCTTACTGCACG-3′
*CBS*	5′-CGCTGCGTGGTCATTCTGCC-3′	5′-TCCCAGGATTACCCCCGCCT-3′
*MPST*	5′-CCAGGTACCGTGAACATCCC-3′	5′-TGTACCACTCCACCCAGGA-3′
*TST*	5′-CCCTTCTCGAAGCCATCCTC-3′	5′-CCAGCTGGTGGATTCAAGGT-3′
*MKI67 (Ki67)*	5′-TCCTTTGGTGGGCACCTAAGACCTG-3′	5′-TGATGGTTGAGGTCGTTCCTTGATG-3′

^1^ Reference gene.

## Data Availability

The datasets used and/or analyzed during the current study are available from the corresponding authors on reasonable request.

## Supplementary Materials

The following supporting information can be downloaded at: https://www.mdpi.com/article/10.3390/cells15020167/s1. Figure S1. Effect of NaCl on *Twist* and *IL-6* mRNA expression in human gastric fibroblasts. qPCR analysis of *Twist* and *IL-6* mRNA expression in non-infected human gastric fibroblasts (Fib) treated daily with 50 µM or 400 µM NaCl for 96 h. Gene expression levels were normalized to the reference gene (*18S* rRNA) and are presented as fold change relative to untreated fibroblasts (Fib). Data are shown as mean ± SEM. No statistically significant changes in *Twist* or *IL-6* mRNA expression were observed following NaCl treatment, indicating that low-dose NaCl does not affect basal expression of these *Hp*-responsive fibroblast activation markers. Results are mean ± SEM of 5 independent experimental repeats. Asterisk (*) indicates a significant change (*p* < 0.05) as compared to the control value (fibroblasts). Figure S2. PCR amplification was performed to determine the presence of *vacA m2* (352 bp), *vacA m1* (290 bp), *vacA s1/s2* alleles (259/286 bp), and *cagA* (298 bp) in multiple *Hp* isolates (lanes 4F-533) and reference strains ATCC 43504 and 700824 (boxed in red). Bands of expected size confirm allele-specific amplification and illustrate strain-dependent virulence genotypes. The ATCC 43504 reference strain shows clear positivity for *vacA m1*, *vacA s1* and *cagA*, consistent with its well-defined virulence profile. We have chosen two *cagA+vacA+* (*s1*/*m1*) positive strains, from which we have further chosen for experiments 43504 strain which induced the strongest fibroblast activation [6,7,8,40,59]. The *Hp* strain negative for *CagA* and *VacA* (*Hp* cagA−vacA− (s2/m2)) did not induce gastric fibroblast activation [40]. Table S1. Primer list.

## Abbreviations

The following abbreviations are used in this manuscript:

3-MST	3-mercaptopyruvate sulfurtransferase
AurkB	aurora kinase B
AzMC	7-Azido-4-Methylcoumarin
CAFs	cancer associated fibroblasts
CBS	cystathionine β-synthase
CSCs	cancer stem cells
CSE	cystathionine γ-lyase
DADS	diallyl disulfide
DYm	mitochondrial membrane potential
EMT-3	epithelial–mesenchymal transition type 3
GC	gastric cancer
GFs	growth factors
GSH	glutathione
H_2_S	hydrogen sulfide
H_2_Sn	polysulfides
Hp	Helicobacter pylori
IKKα	IκB kinase α
IKKβ	IκB kinase beta
MBC	minimum bactericidal concentration
NHGFs	normal human gastric fibroblasts
Nrf2	nuclear-factor-E2-related factor-2
Rb	retinoblastoma protein
ROS	reactive oxygen species
RSS	reactive sulfur species
SOP	sulfur oxidation pathway
SQR	sulfide quinone reductase
SSS	sulphane sulfur species
STS	sodium thiosulfate
TCGA	The Cancer Genome Atlas
TME	tumor microenvironment
WB	Western blot
