# Supplementary material for: Hydrogen Sulfide Inhibits H. pylori-Induced Gastric Fibroblast Activation: Implications for Cancer Prevention
Source: Cells. 2026 Jan 16;15(2):167. doi: 10.3390/cells15020167 (PMC12839146; doi:10.3390/cells15020167)
Supplement: Supplementary file 1 [file cells-15-00167-s001.zip › cells-3995425-supplementary.pdf]

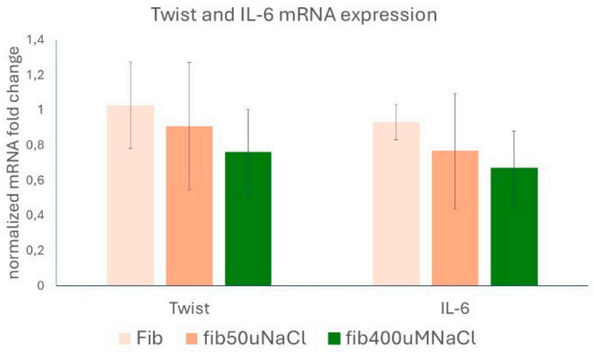

Supplementary Figure S1. Effect of NaCl on Twist and IL-6 mRNA expression in human gastric fibroblasts. qPCR analysis of Twist and IL-6 mRNA expression in non-infected human gastric fibroblasts (Fib) treated daily with 50 µM or 400 µM NaCl for 96hrs. Gene expression levels were normalized to the reference gene (18S rRNA) and are presented as fold change relative to untreated fibroblasts (Fib). Data are shown as mean ± SEM. No statistically significant changes in Twist or IL-6 mRNA expression were observed following NaCl treatment, indicating that low-dose NaCl does not affect basal expression of these Hp-responsive fibroblast activation markers. Results are mean ± SEM of 5 independent experimental repeats. Asterisk (*) indicates a significant change (P<0.05) as compared to the control value (fibroblasts).

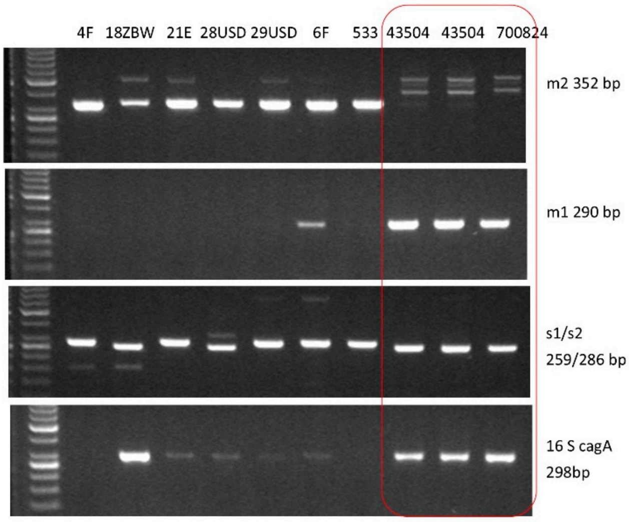

Supplementary Figure S2.

PCR amplification was performed to determine the presence of vacA m2 (352 bp), vacA m1 (290 bp), vacA s1/s2 alleles (259/286 bp), and cagA (298 bp) in multiple Hp isolates (lanes 4F-533) and reference strains ATCC 43504 and 700824 (boxed in red). Bands of expected size confirm allele-specific amplification and illustrate strain-dependent virulence genotypes. The ATCC 43504 reference strain shows clear positivity for vacA m1, vacA s1 and cagA, consistent with its well-defined virulence profile. We have chosen two cagA+vacA+ (s1/m1) positive strains, from which we have further chosen for experiments 43504 strain which induced the strongest fibroblast activation [6-8,40,59]. The Hp strain negative for CagA and VacA (Hp cagA−vacA− (s2/m2) did not induce gastric fibroblast activation [40].

Primer list

Gene	Forward Primer Sequence	Reverse Primer Sequence
cagA	5′-ATAATGCTAAATTAGACAACTTGAGCG-3′	5′-TTAGAATAATCAACAAACATCACGCCA-3′
vacA m1	5′-GGTCAAAATGCGGTCATGG-3′	5′-CCATTGGTACCTGTAGAAAC-3′
vacA m2F	5′GGAGCCCCAGGAAACATTG-3′	5′-CATAACTAGCGCCTTGCAC-3′
vacA s1/s2	5′-ATGGAAATACAACAAACACAC-3′	5′-CTGCTTGAATGCGCCAAAC-3′
